# High levels of endemism and local differentiation in the fungal and algal symbionts of saxicolous lecideoid lichens along a latitudinal gradient in southern South America

**DOI:** 10.1017/S0024282920000225

**Published:** 2020-07

**Authors:** Ulrike Ruprecht, Fernando Fernández-Mendoza, Roman Türk, Alan M. Fryday

**Affiliations:** 1 Universität Salzburg, FB Biowissenschaften, Hellbrunnerstrasse 34, 5020 Salzburg, Austria; 2 Karl-Franzens-Universität Graz, Institut für Biologie, Holteigasse 6, 8010 Graz, Austria; 3 Department of Plant Biology, Michigan State University, East Lansing, MI 48824, USA

**Keywords:** glacial refugia, global distribution, pioneer vegetation on rock, subantarctic subregion

## Abstract

Saxicolous, lecideoid lichenized fungi have a cosmopolitan distribution but, being mostly cold adapted, are especially abundant in polar and high-mountain regions. To date, little is known of their origin or the extent of their trans-equatorial dispersal. Several mycobiont genera and species are thought to be restricted to either the Northern or the Southern Hemisphere, whereas others are thought to be widely distributed and occur in both hemispheres. However, these assumptions often rely on morphological analyses and lack supporting molecular genetic data. Also unknown is the extent of regional differentiation in the southern polar regions. An extensive set of lecideoid lichens (185 samples) was collected along a latitudinal gradient at the southern end of South America. Subantarctic climate conditions were maintained by increasing the elevation of the collecting sites with decreasing latitude. The investigated specimens were placed in a global context by including Antarctic and cosmopolitan sequences from other studies. For each symbiont three markers were used to identify intraspecific variation (mycobiont: ITS, mtSSU, *RPB1*; photobiont: ITS, psbJ-L, COX2). For the mycobiont, the saxicolous genera *Lecidea*, *Porpidia*, *Poeltidea* and *Lecidella* were phylogenetically re-evaluated, along with their photobionts *Asterochloris* and *Trebouxia*. For several globally distributed species groups, the results show geographically highly differentiated subclades, classified as operational taxonomical units (OTUs), which were assigned to the different regions of southern South America (sSA). Furthermore, several small endemic and well-supported clades apparently restricted to sSA were detected at the species level for both symbionts.

## Introduction

Saxicolous ‘lecideoid’ lichens (Hertel 1984) are genera and species usually described originally under the generic name *Lecidea sensu* Zahlbruckner (1925) and comprise crustose species with apothecia lacking a thalline margin and with non-sepate ascospores. As such they are a heterogenous, non-monophyletic group and, although some do belong to the genus *Lecidea* s. str. (*Lecideaceae* Chevall.), most belong to other genera and/or families, such as *Porpidia* Körb, *Poeltidea* Hertel & Hafellner and *Cyclohymenia* McCune & M. J. Curtis (*Lecideaceae*), *Carbonea* (Hertel) Hertel and *Lecidella* Körb. (*Lecanoraceae* Körb.). In addition to their morphological similarities, lecideoid lichenized fungi are strongly associated with green microalgal photobionts of the cosmopolitan class Trebouxiophyceae (Hertel 1984, 2007; Buschbom & Mueller 2004; Schmull *et al.* 2011; Ruprecht *et al.* 2012*a*, 2016; Fryday & Hertel 2014; Zhao *et al.* 2015).

The family *Porpidiaceae* was erected by Hertel & Hafellner (Hafellner 1984) to accommodate the genus *Porpidia* along with other genera formerly included in the *Lecideaceae* that had a *Porpidia-*type ascus structure. However, Buschbom & Mueller (2004) showed that *Lecidea* was phylogenetically nested within the *Porpidiaceae* and so the latter family was included in the synonymy of the *Lecideaceae* (*Lecideales*) by Lumbsch & Huhndorf (2010), and this is still the case in the current issue of *Outline of Ascomycota: 2017* (Wijayawardene *et al.* 2018). The genera *Lecidea* s. str. (Hertel 1984), *Porpidia* and *Poeltidea* as well as *Cyclohymenia* and *Farnoldia* Hertel are all assigned to this family, although *Farnoldia* appears to occupy a peripheral position. The other genus investigated here, *Lecidella* was also originally included in the *Lecideaceae* but the species of this genus have an ascus structure very similar to the *Lecanora*-type and so the genus was included in the *Lecanoraceae* by Hafellner (1984), which is still its current position (Wijayawardene *et al.* 2018).

The inconspicuous morphology of ‘lecideoid’ lichens complicates their systematic treatment. Large taxonomic groups are often distinguishable by just a small number of microscopic traits, such as spore size and septation or ascus-type, but species-level identification can be difficult, often relying on subtle characters such as excipulum pigmentation and structure or the secondary metabolites produced. The fundamental taxonomic work on lecideoid lichens (Hafellner 1984; Hertel 1984; Gowan 1989; Knoph & Leuckert 1994; Inoue 1995; Castello 2003; Knoph *et al.* 2004; Fryday 2005; Fryday & Hertel 2014) has mostly used morphological and chemical characters, but lacks molecular genetic data. Extensive collections, especially from the Southern Hemisphere, are very often older than 50 years which precludes the use of molecular methods because of the common problem of DNA degradation in mycobiont specimens older than 20 years. During the last decade, molecular re-evaluations have helped to redefine the species concepts behind these diverse groups but were mostly focused on the Northern Hemisphere (Buschbom & Mueller 2004; Schmull *et al.* 2011; Orange 2014; Zhao *et al.* 2015, 2016; McCune *et al.* 2017) and Antarctica (Ruprecht *et al.* 2010, 2012*b*). However, Hale *et al.* (2019) recently demonstrated a biogeographical connection between the *Lecidea* species of western North America and the southern polar regions, helping to provide a better understanding of distribution and speciation patterns in this group. Nevertheless, intermediate latitudes in the Southern Hemisphere remain understudied and recently published results (Ruprecht *et al.* 2016) have emphasized the extent of the knowledge gap in southern South American lecideoid lichens, not only from the mycobiont perspective but also from that of the associated green microalgae.

The use of DNA sequence data and phylogenetic methods has revealed that cosmopolitan genera often show locally differentiated subgroups or cryptic species, which can be influenced by ecological factors and may be restricted to isolated areas (Walser *et al.* 2005; Leavitt *et al.* 2011; Lumbsch & Leavitt 2011; Branco *et al.* 2015; Kraichak *et al.* 2015). Lichens, as well as non-lichenized fungi, with an arctic-alpine distribution in the Northern Hemisphere are, however, a notable exception to this pattern, often comprising relatively homogenous genetic entities, mostly at the species level, with widespread distributions. A number of studies, such as those on *Porpidia flavicunda* (Ach.) Gowan (Buschbom 2007), *Flavocetraria cucullata* (Bellardi) Kärnefelt & A. Thell and *F. nivalis* (L.) Kärnefelt & A. Thell (Geml *et al.* 2010) as well as for several different types of fungi (Geml 2011), indicate continuing intercontinental gene-flow in species that are present in both the Northern and Southern Hemispheres. However, trans-equatorial dispersal is also shown for other, similar lineages, such as the lichenized fungal genus *Lichenomphalia* Redhead *et al.* (Geml *et al.* 2012) or the species *Cetraria aculeata* (Schreb.) Fr. (Fernández-Mendoza & Printzen 2013). For the algal partners, although the distribution of green-algal photobionts of the genus *Trebouxia* Puymaly extends across broad intercontinental regions, especially in the Northern Hemisphere (Leavitt *et al.* 2015), a pattern of trans-equatorial dispersal with low diversification is common (Muggia *et al.* 2010; Ruprecht *et al.* 2012*a*), with one exception in the most extreme areas of continental Antarctica (Ruprecht *et al.* 2012*a*). However, for the mycobionts, strong diversification and endemism in the Southern Hemisphere is expected based on morphology, resulting in the development of several distinct species and genera, such as *Lecidea aurantia* Fryday, *L. cambellensis* Fryday (Fryday & Hertel 2014) or *L. cancriformis* C. W. Dodge & G. E. Baker (Castello 2003), *Poeltidea* (Hertel 1984), *Gondwania* Søchting *et al.*, *Shackletonia* Søchting *et al.* (Arup *et al.* 2013) and *Protousnea* (Motyka) Krog. (Calvelo *et al.* 2005).

The most probable scenarios for disjunct distributions are that they can be caused 1) by vicariance and mid-distance dispersal, as shown in the genus *Chroodiscus* (Lücking *et al.* 2008) through the vicinity and interconnection of continental shelves, or 2) by transition from the Arctic to Patagonia in the Pleistocene, resulting in cryptic specialization, as shown in the bipolar lichen *Cetraria aculeata* (Fernández-Mendoza & Printzen 2013), or 3) by glacial refugia during the last ice ages at the southern end of South America (Paula & Leonardo 2006). A good example of this last scenario is the highly differentiated and endemic lichen species *Porpidia navarina* U. Rupr. & Türk, which is known only from one of the southernmost islands (Isla Navarino) that was ice free during the Last Glacial Maximum (Douglass *et al.* 2005; Ruprecht *et al.* 2016). Additionally, evolutionary processes such as adaptation and subsequent specialization to the harsh climate conditions in Antarctic cold deserts (Ruprecht *et al.* 2010, 2012*b*; Schroeter *et al.* 2011) can also lead to high local differentiation in global species and endemism in the southern polar regions.

Lichens are ideal model-systems to test these hypotheses because several genera and species are globally distributed and form locally differentiated subgroups (Fernández-Mendoza *et al.* 2011). Additionally, at least double the information is available compared to other organisms because lichens consist of a symbiotic relationship between two or more independently distributed partners. This main symbiotic relationship is formed by a fungus (mycobiont) and green algae and/or cyanobacteria (photobiont). Furthermore, a diverse community of associated bacteria (Grube *et al.* 2015; Aschenbrenner *et al.* 2016), algae (Peksa & Škaloud 2011; Ruprecht *et al.* 2014; Moya *et al.* 2017), endolichenic or lichenicolous fungi and basidiomycete yeasts (Lawrey & Diederich 2003; Arnold *et al.* 2009; Spribille *et al.* 2016) are part of the lichen thallus.

**Fig. 1.**
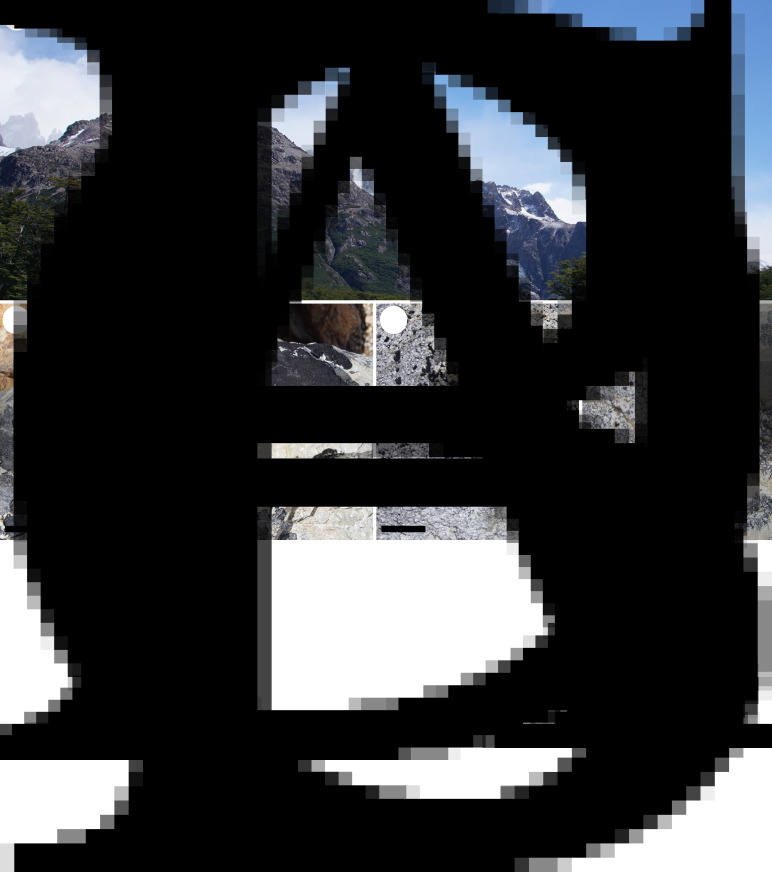
A, classical subantarctic subregion above tree level: Parque Nacional Los Glaciares, Argentina. B, saxicolous crustose lichens on siliceous rock, *Lecidea auriculata*, *L. kalbii*, *Poeltidea perusta*, *Rhizocarpon geographicum*. C, *Lecidea lapicida*. D, *Poeltidea* sp. 1. Scales: B = 10 cm; C & D = 10 mm. In colour online.

This study focuses on the geographically isolated, tapering southern end of the South American continent (southern Patagonia, including the islands around Tierra del Fuego and Cape Horn). Due to climatic conditions equivalent to Maritime Antarctica, the southern subpolar region (or subantarctic subregion, which is characterized by an absence of arboreal vegetation and is located between the Antarctic Divergence and Subtropical Convergence: Morrone 2000; Brummitt 2001) is included as part of the Antarctic floral kingdom (Takhtajan & Cronquist 1986). The subantarctic subregion extends northwards through the continent at increasing elevations along the mountain ranges of the southern Andes (Morrone 2000). To the south, Maritime Antarctica is the closest landmass, separated by *c*. 900 km of ocean and the Antarctic Circumpolar Current (Allison *et al.* 2010; McCave *et al.* 2014). These areas are colonized by specialized cold-adapted organisms, which often act as pioneer vegetation (Hertel 1984; Caccianiga & Andreis 2004; Bilovitz *et al.* 2015). Among other organisms, the globally distributed saxicolous lecideoid lichens are one of the most frequent components of the biota, forming diverse communities on rocks and boulders, mainly in treeless areas above a temperate rainforest (Fig. 1; Hertel 2007; Goffinet *et al.* 2012; Ruprecht *et al.* 2016).

In the current study, we wish to explore the genetic diversity of the two dominant symbionts (myco- and photobiont), along with their distribution and diversification along a latitudinal gradient in southern South America. These were newly estimated with three marker datasets for each symbiont, based on both tree-based and non-tree based clustering methods, including distance-based clustering, model-based phylogenies and coalescent analyses. To accomplish this, both symbionts of saxicolous lecideoid lichen specimens from southern South America were placed in a global context using sequence information from the first author's project framework, from collaborating partners and from international databases (e.g. GenBank). Additionally, newly generated sequences of species that had previously only been described morphologically were included, as well as sequences of uncertain position in the genera *Lecidea* s. str. (Hertel 1984), *Porpidia*, *Poeltidea*, *Lecidella* (mycobiont) and *Asterochloris* Tschermak-Woess and *Trebouxia* (photobiont).

## Material and Methods

### Collecting sites and material

One hundred and eighty-five saxicolous lecideoid lichen samples were collected in southern South America along a latitudinal gradient following the subantarctic climatic subregion by increasing elevation from south (Cerro Bandera, Isla Navarino, Chile, 55°S, 620 m a.s.l.) to north (Cerro Catedral, Bariloche, Argentina, 41°S, 2100 m a.s.l.) and including some areas at a lower elevation in southern Chile.

**Fig. 2.**
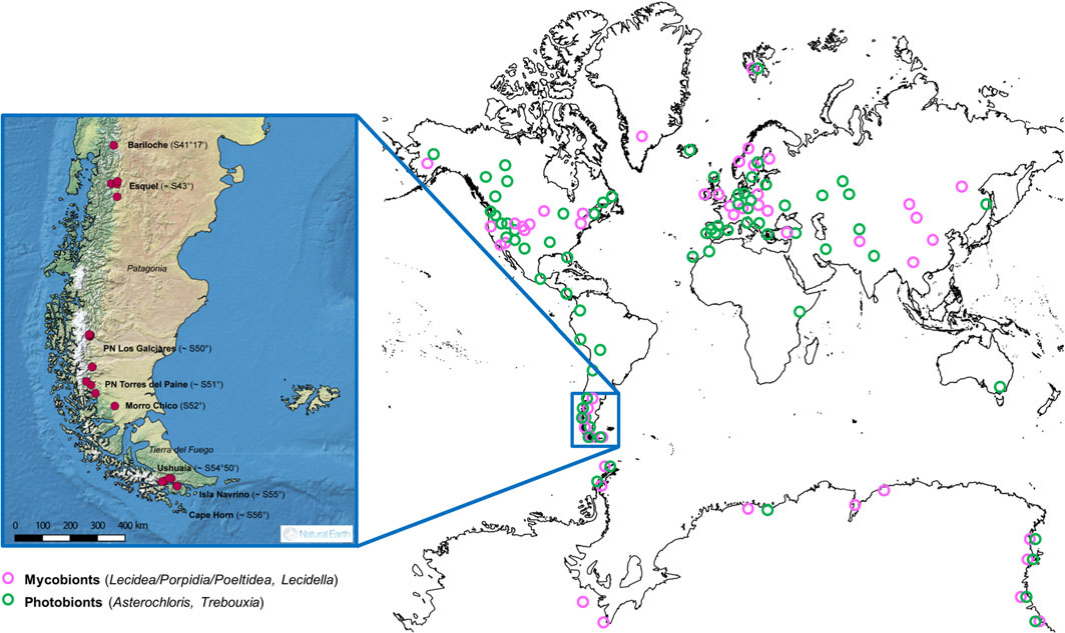
World map showing the locations of the included accessions obtained from GenBank and from our own database. Pink circles show the collection points of the mycobiont and green circles of the photobiont accessions. The enlarged map (inset) shows the sampling sites from this study in southern South America.

The specimens were collected from siliceous rock in areas above the tree line that were dominated by subantarctic climatic conditions with an annual mean temperature (BIO_1_) of 0 to 7.8 °C and an annual precipitation (BIO_12_) of 320 to 1640 mm (Karger *et al*. 2017; Fig. 2, Supplementary Material Files S1-1 & S1-2a, available online).

All our specimens collected from southern South America (sSA) are deposited in the Herbarium of the University of Salzburg (SZU).

### DNA amplification and sequencing

Total DNA was extracted from individual thalli using the DNeasy Plant Mini Kit (Qiagen) according to the manufacturer's instructions. The lichen material was scraped off with a sterilized scalpel from the centre of the thallus and included apothecia. The PCR mix contained 0.5 units of GoTaq DNA polymerase, 0.2 nM of each of the four dNTPs, 0.3 μM of each primer and *c*. 1 ng genomic DNA.

For each symbiont, three markers were amplified and sequenced with the primers presented in Supplementary Material File S1-3, with conditions as described in Ruprecht *et al.* (2014) and Ruprecht *et al.* (2016). Unpurified PCR products were sent to Eurofins Genomics/Germany for sequencing.

#### 

##### Mycobiont

The internal transcribed spacer region of the nuclear ribosomal DNA (ITS) was amplified for all specimens. Furthermore, the mitochondrial small subunit (mtSSU) and the large subunit of DNA-dependent RNA polymerase 2 (*RPB1*) were amplified for the *Lecidea/Porpidia/Poeltidea* group (see Supplementary Material File S1-3).

##### Photobiont

To obtain a first overview of the different taxa at species and/or genus level of the associated green microalgae available to the mycobiont, a broad screening along the internal transcribed spacer region of the nuclear ribosomal DNA (ITS) was performed using the different primer pairs ITS1T and ITS4T (Kroken & Taylor 2000), ITS-sense-A and ITS-antisense-A (Ruprecht *et al.* 2014) and NS7m and LR1850 (Bhattacharya *et al.* 1996) as described in Ruprecht *et al.* (2016). Also, for *Trebouxia* a chloroplast marker, a variable protein-coding gene including an intergenic spacer region (psbJ-L), and a fragment of the mitochondrial cytochrome oxidase subunit 2 gene (COX2) were chosen (Supplementary Material File S1-3). Additionally, all three markers were sequenced from four *Trebouxia* cultures (2 × S02/Antarctica, A02/Antarctica and A12/Sweden; Supplementary Material File S1-2d) provided by S. Ott (Düsseldorf), to calibrate the concatenated dataset.

### Phylogenetic analyses

Sequences were assembled and edited using Geneious Pro 6.1.8 (www.geneious.com), and aligned with MAFFT v7.017 (Katoh *et al.* 2002) using pre-set settings (algorithm, auto select; scoring matrix, 200PAM/k = 2; gap open penalty, 1.34–0.123).

Maximum likelihood analyses (ML) were performed using the IQ-TREE web server (Trifinopoulos *et al.* 2016) with default settings (ultrafast bootstrap analyses, 1000 BT alignments, 1000 max. iterations, min. correlation coefficient: 0.99, SH-aLRT branch test with 1000 replicates) and presented as a consensus tree. The respective evolutionary models were selected with the implemented model finder (Kalyaanamoorthy *et al.* 2017) of the program IQ-TREE (Supplementary Material File S1-4).

The Bayesian phylogenies were inferred using the Markov chain Monte Carlo (MCMC) procedure as implemented in the program MrBayes 3.2 (Ronquist & Huelsenbeck 2003). The analysis was performed assuming the general time reversible model of nucleotide substitution including estimation of invariant sites and a discrete gamma distribution with six rate categories (GTR + I + Γ; Rodriguez *et al.* 1990). Two runs with 5 million generations, each starting with a random tree and employing four simultaneous chains, were executed. Every 1000th tree was saved into a file. Subsequently, the first 25% of trees was deleted as the ‘burn-in’ of the chain. A consensus topology with posterior probabilities for each clade was calculated from the remaining 3751 trees. The phylogenies of the mycobiont of the *Lecidea/Porpidia/Poeltidea* group were rooted with *Farnoldia jurana* subsp. *jurana* (Schaer.) Hertel, and for *Lecidella* with species of the closely related genera *Lecanora* Ach., *Rhizoplaca* Zopf and *Carbonea*; the algal phylogenies were midpoint rooted. All phylogenies were visualized with the program Figtree v1.4.3 (Rambaut 2014).

### OTU- and cluster delimitation

For each phylogenetically coherent group (*Lecidea/Porpidia/Poeltidea*, *Lecidella*, *Asterochloris* and *Trebouxia*), the ITS marker was used to generate operational taxonomical units (OTUs) using automatic barcode gap discovery (ABGD; Puillandre *et al.* 2012) to additionally define the well-supported intraspecific (cryptic) subgroups with a distance-based method. The default settings were used, except that ‘X’ (relative gap) = 0.9 and the distance JC69 were chosen. OTUs with a sequence similarity lower than 97.5% were divided into subunits using phylogenetic criteria based on Leavitt *et al.* (2015) for *Trebouxia* (Supplementary Material File S1-5).

An alternative cluster delimitation specifically looking for boundaries between population and species-level processes was implemented by a recursive multi-tree application of the general mixed Yule-coalescent model (bGMYC; Pons *et al.* 2006; Reid & Carstens 2012; Fujisawa & Barraclough 2013) on the ITS phylogeny of *Lecidea/Porpidia/Poeltidea* to define species-clades and/or species-complexes on a higher scale. For this purpose, substitution model adequacy was estimated using ML reconstructions in IQ-TREE (Minh *et al.* 2013; Kalyaanamoorthy *et al.* 2017), resulting in the selection of a TIM2 transition model with empirical frequencies and a Ratefree (Yang 1995; Soubrier *et al.* 2012) model with 4 relaxed gamma categories. Time explicit phylogenetic reconstructions were carried out in Beast v2.5.1 (Bouckaert *et al.* 2014) using a strict clock, the suitability of which was previously assessed in MEGA (Tamura *et al.* 2011), and a constant size coalescent prior, which is the most conservative tree model in terms of including false positives. A maximum clade credibility tree was calculated in TreeAnnotator (Bouckaert *et al.* 2014) using most recent common ancestor (mrca) dates. The bGMYC analyses were carried out as implemented in the homonymous R package. A single GMYC analysis was iteratively run on a subset of 1000 trees chosen randomly from the posterior distribution, using a chain length of 50 000 sampling steps, a burn-in of 40 000 and a thinning parameter of 100. The results of all GMYC analyses are summarized in a matrix of pairwise co-assignment probabilities for each haplotype. To obtain a consensus partition we processed the co-assignment matrix using an arbitrarily chosen co-assignment threshold of 0.5, and a less arbitrary approach making use of k-medoid clustering (Kaufman & Rousseeuw 1990) and optimum average silhouette width as validation criterion to estimate the optimum number of clusters (Ortiz-Alvarez *et al.* 2015). For the latter we used the function *pamk* as implemented in the R package ‘fpc’ (Henning 2014) on the co-assignment matrix converted into its dissimilarity correlate. Clustering validation was carried out using the function clValid from the homonymous R package (Brock *et al.* 2008); the discrepancy between stability metrics highlights the discrepancy between the number of clusters and the number of sequences per cluster.

## Results

### Phylogenetic analyses, OTU and cluster delimitation

**Fig. 3.**
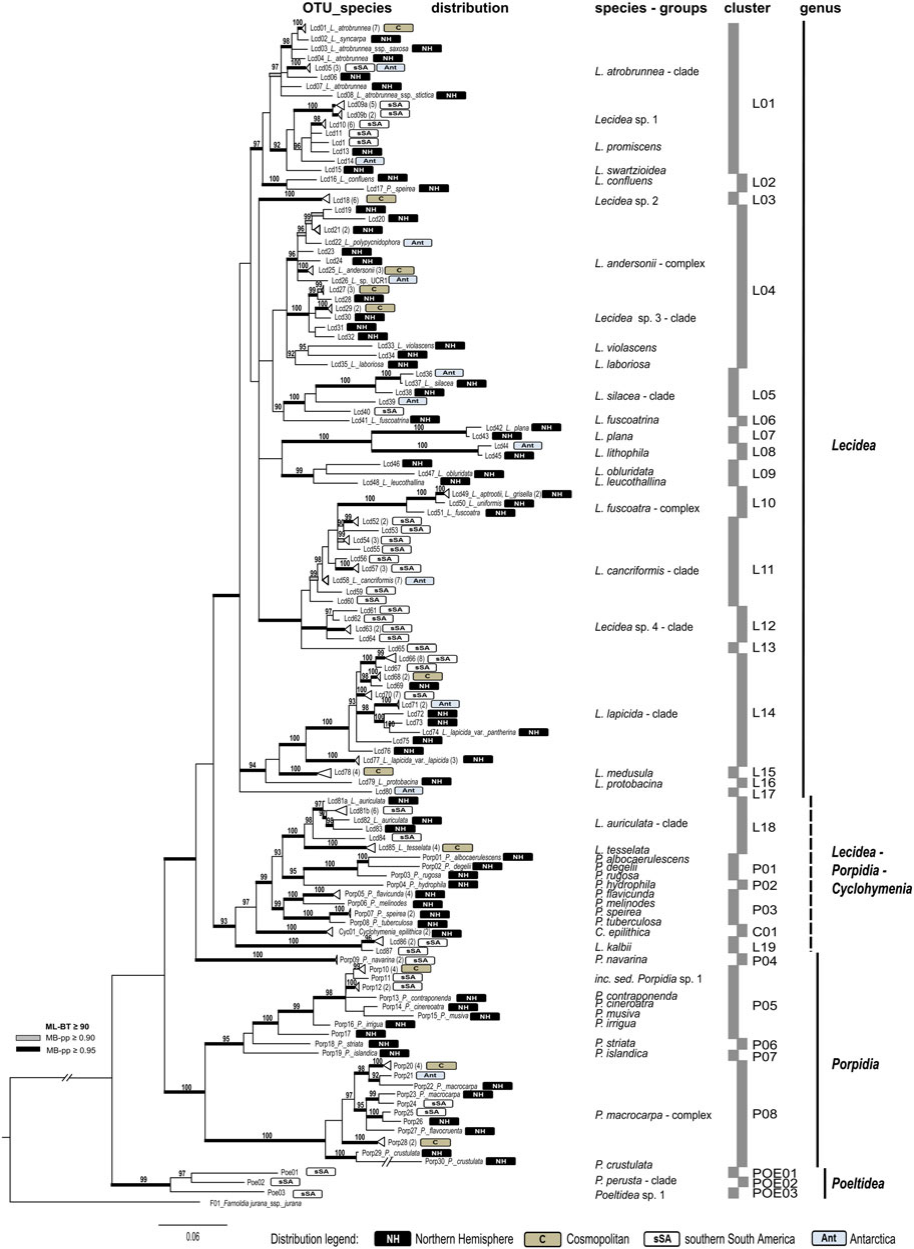
Collapsed phylogeny on OTU-level including all available relevant taxonomically identified sequences of the genera *Lecidea, Porpidia*, *Poeltidea* and *Cyclohymenia* using the marker ITS. Voucher information included in the OTUs is provided in Supplementary Material File S2-1a and the complete tree is shown in S2-1b (available online). Further information is also available in Supplementary Material Files S1-2a & S1-2c. Alphanumeric codes represent OTUs and numbers in brackets indicate the number of sequences comprising that OTU (see also Supplementary Material File S1-5). The biogeographic distribution (NH, Northern Hemisphere; C, cosmopolitan; sSA, southern South America; Ant, Antarctica) has been added beside the OTUs. New sequences of specimens from sSA are in bold italics and those from other parts of the world are in bold. The vertical bars beside the phylogeny show the affiliation to clusters and genera. The bootstrap values with ≥ 95 support of ML analyses were directly mapped on the Bayesian tree with ≥ 0.90 (grey) and ≥ 0.95 (black) support posterior probability values (branches in bold). In colour online.

**Fig. 4.**
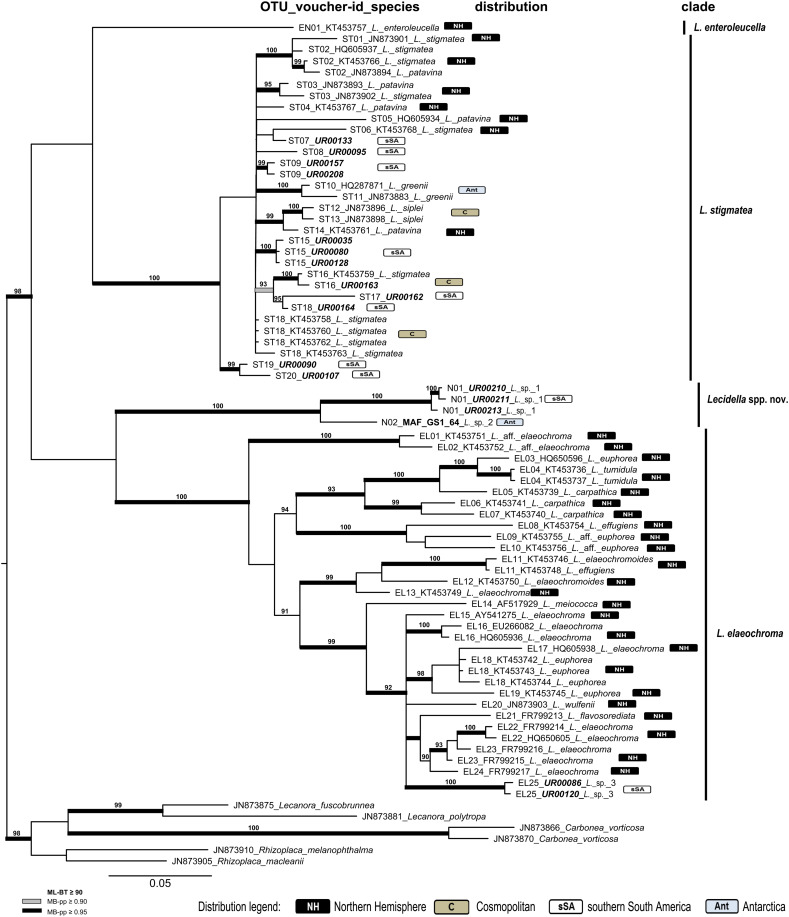
Phylogeny of the genus *Lecidella* with accessions from sSA (see Supplementary Material File S1-2a, available online) integrated in the species concept and most of the published accessions (Zhao *et al.* 2015; see also Supplementary Material File S1-2c) using the marker ITS. OTU numbers precede voucher numbers and the published species names from GenBank, as well as the biogeographic distribution information, for each OTU is included (NH, Northern Hemisphere; C, cosmopolitan; sSA, southern South America; Ant, Antarctica). New sequences of specimens from sSA are in bold italics and those from other parts of the world are in bold. The vertical bars beside the phylogeny show the affiliation to the clades. The bootstrap values with ≥ 95 support of ML analyses were directly mapped on the Bayesian tree with ≥ 0.90 (grey) and ≥ 0.95 (black) support posterior probability values (branches in bold). In colour online.

**Fig. 5.**
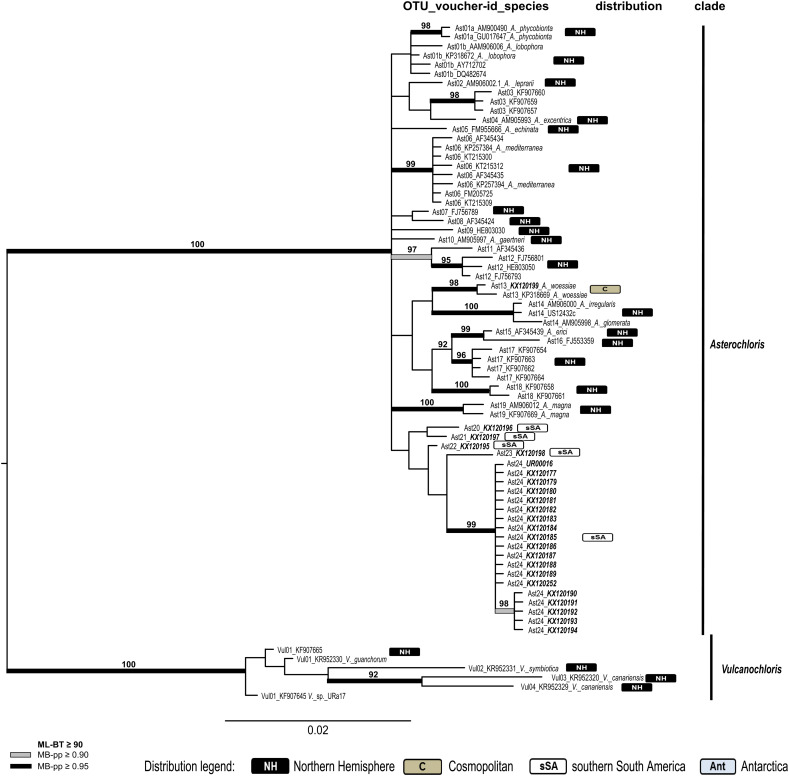
Phylogeny of the genus *Asterochloris* including all available relevant taxonomically identified sequences using the marker ITS (see Supplementary Material Files S1-2a & S1-2c, available online). OTU numbers precede voucher numbers and the published species names from GenBank as well as the biogeographic distribution information for each OTU is included (NH, Northern Hemisphere; C, cosmopolitan; sSA, southern South America; Ant, Antarctica). New sequences of specimens from sSA are marked in bold italics. The vertical bars beside the phylogeny show the affiliation to the genera. The bootstrap values with ≥ 95 support of ML analyses were directly mapped on the Bayesian tree with ≥ 0.90 (grey) and ≥ 0.95 (black) support posterior probability values (branches in bold). In colour online.

**Fig. 6.**
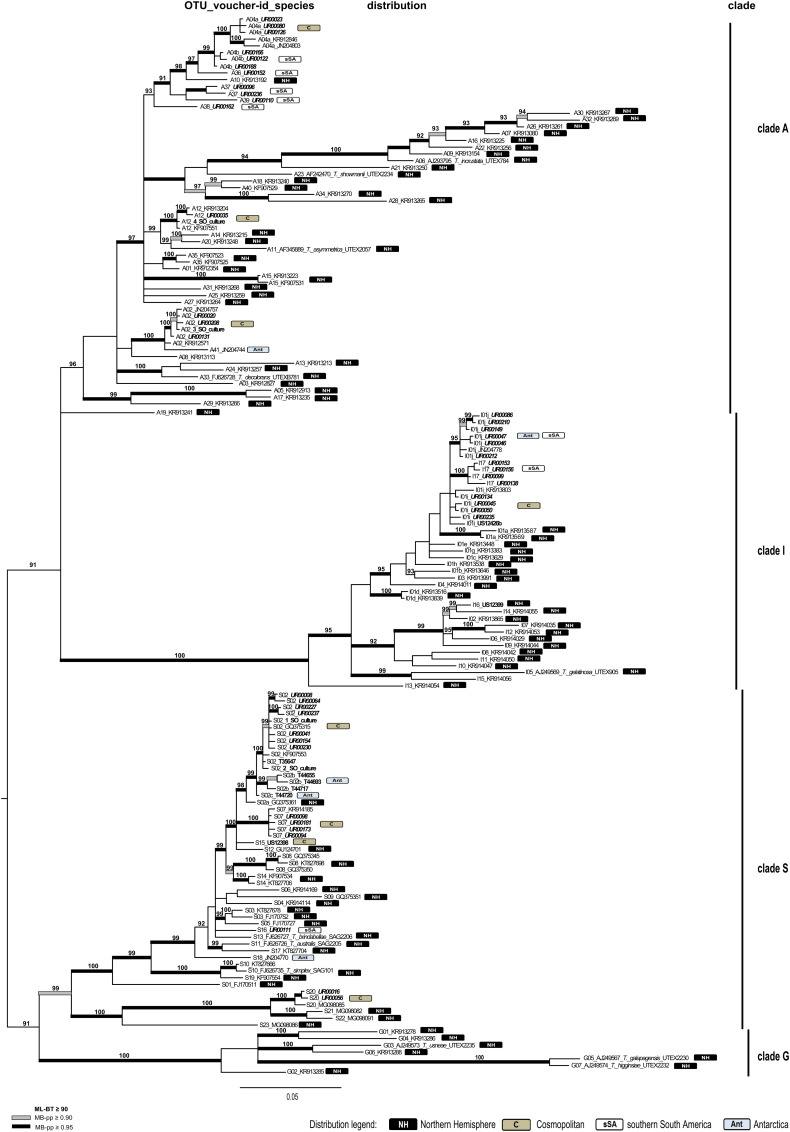
Phylogeny of the genus *Trebouxia* with accessions from sSA integrated into the species concept and most of the published accessions of Leavitt *et al.* (2015) using the marker ITS (see Supplementary Material Files S1-2b & S1-2d, available online). OTU numbers precede voucher numbers and the published species names from GenBank as well as the biogeographic distribution information for each OTU is included (NH, Northern Hemisphere; C, cosmopolitan; sSA, southern South America; Ant, Antarctica). New sequences of specimens from sSA are in bold italics and those from other parts of the world are in bold. The vertical bars beside the phylogeny show the affiliation to the clades. The bootstrap values with ≥ 95 support of ML analyses were directly mapped on the Bayesian tree with ≥ 0.90 (grey) and ≥ 0.95 (black) support posterior probability values (branches in bold). In colour online.

Four overall phylogenies for the saxicolous genera *Lecidea/Porpidia/Poeltidea* (*Lecideaceae*), *Lecidella* (*Lecanoraceae*), *Asterochloris* and *Trebouxia* (Trebouxiophyceae) were estimated using the ITS marker (Figs 3–6, Supplementary Material Files S2-1a & b, available online). Additionally, two multi-marker trees for *Lecidea/Porpidia/Poeltidea* (ITS/mtSSU/*RPB1*; Supplementary Material File S2-2) and *Trebouxia* (ITS/psbJ-L/COX2; Supplementary Material File S2-3) were also estimated.

In all cases the ML analyses (IQ-TREE web server; Trifinopoulos *et al.* 2016) of the six phylogenies recovered the same tree topology as the MrBayes analysis (Ronquist & Huelsenbeck 2003). Therefore, we present here only the Bayesian tree with support values of the ML analyses.

For the multi-marker trees, no conflict was found with the single marker trees, thus they are not shown.

OTUs with a sequence similarity lower than 97.5% were divided into subunits using phylogenetic criteria based on Leavitt *et al*. (2015) for *Trebouxia*. This concept was used as analogous to an appropriate delimitation method for cryptic (molecular) diversification on a molecular basis to show the biogeographical distribution of the OTUs (Tables 1–3). These parameters were used for all four overall phylogenies based on the ITS marker. The sequence similarity values of the OTUs with at least three accessions are summarized in Supplementary Material File S1-5. The biogeographical distribution of the locally differentiated OTUs was added beside the OTU, voucher and species information in the figures.

**Table 1.** Geographical distributions of selected mycobiont species and OTUs, arranged in groups according to shared distributions at different levels of clustering. Subtotals of numbers of species, specimens (*n*) and OTUs according to shared distributions are presented, with the total given below. Species and OTUs with different distributions (globally/sSA) are marked in bold. Accessions occurring in Antarctica and sSA are summarized as southern polar. * = species that were found in two different categories and were included only once in the total sum. See Supplementary Material File S1-2a (available online) for further information on the listed specimens.

Species - distribution		*n*	OTU - distribution	
*Lecidea* sp. 2	globally	8	LP_Lcd18	globally
*Lecidea* sp. 3 - clade	globally	4	LP_Lcd27, 29	globally
*Lecidea atrobrunnea -* clade*	globally	3	LP_Lcd01	globally
*Lecidea medusula*	globally	3	LP_Lcd78	globally
*Lecidea tessellata*	globally	3	LP_Lcd85	globally
*Porpidia macrocarpa -* complex*	globally	4	LP_Porp20	globally
Subtotal 6		25	7	
*Lecidea atrobrunnea -* clade*	**globally**	**2**	**LP_Lcd05**	**southern polar**
***Lecidea auriculata -* clade**	**globally**	**14**	**LP_Lcd81b, 83**	**sSA**
***Lecidea lapicida -* clade**	**globally**	**48**	**LP_Lcd66, 67, 70, 75**	**sSA**
***Lecidea promiscens***	**globally**	**9**	**LP_Lcd10 - 12**	**sSA**
***Lecidella stigmatea***	**globally**	**13**	**LL_ST07, 08, 09, 15–20**	**sSA**
***inc. sed. Porpidia* sp. 1**	**globally**	**5**	**LP_Porp11, 12**	**sSA**
*Porpidia macrocarpa -* complex*	**globally**	**2**	**LP_Porp24, 25**	**sSA**
Subtotal 7		93	23	
*Lecidea* sp. 1	sSA	8	LP_Lcd09a, 09b	sSA
*Lecidea* sp. 4 - clade	sSA	8	LP_Lcd61 - 64	sSA
*Lecidella* sp. 1	sSA	3	LL_N01	sSA
*Lecidella* sp. 3	sSA	2	LL_EL25	sSA
*Poeltidea* sp.1	sSA	3	LP_Poe03	sSA
*Lecidea cancriformis -* clade	southern polar	19	LP_Lcd52 - 57, 59, 60	sSA
*Lecidea kalbii*	sSA	4	LP_Lcd86, 87	sSA
*Lecidea* sp. UR00096	sSA	1	LP_Lcd65	sSA
*Lecidea* sp. UR00130	sSA	1	LP_Lcd40	sSA
*Porpidia navarina*	sSA	13	LP_Porp09	sSA
*Poeltidea perusta*	sSA	5	LP_Poe01, 02	sSA
Subtotal 12		67	24	
Total 23		185	54

**Table 2.** Geographical distributions of selected photobiont species and OTUs, arranged in groups according to shared distributions at different levels of clustering. Subtotals of numbers of specimens (*n*) and OTUs according to shared distributions are presented, with the total given below. See Supplementary Material File S1-2a (available online) for further information on the listed specimens.

Species	*n*	OTU - distribution	
*Asterochloris woessiae*	1	Ast13	globally
*Trebouxia* sp.	7	Tr_A02	globally
*Trebouxia* sp.	5	Tr_A04a	globally
*Trebouxia* sp.	6	Tr_A12	globally
*Trebouxia* sp.	5	Tr_I01i	globally
*Trebouxia* sp.	84	Tr_S02	globally
*Trebouxia* sp.	17	Tr_S07	globally
*Trebouxia* sp.	3	Tr_S20	globally
Subtotal	128	8	
*Asterochloris* sp. UR00027	1	Ast22	sSA
*Asterochloris* sp. UR00123	1	Ast21	sSA
*Asterochloris* sp. URa18	19	Ast24	sSA
*Asterochloris* sp. URa19	1	Ast23	sSA
*Trebouxia* sp.	6	Tr_A04b	sSA
*Trebouxia* sp.	1	Tr_A36	sSA
*Trebouxia* sp.	2	Tr_A37	sSA
*Trebouxia* sp.	4	Tr_A38	sSA
*Trebouxia* sp.	5	Tr_A39	sSA
*Trebouxia* sp.	20	Tr_I01j	southern Polar
*Trebouxia* sp.	11	Tr_I17	sSA
*Trebouxia* sp.	3	Tr_S16	sSA
Subtotal	74	12	
Total	202	20

**Table 3.** Summary of sampling sites in southern South America (sSA) with latitude, altitude, climate variables BIO 1 (annual mean temperature) and BIO 12 (annual precipitation) using CHELSA (Karger *et al.* 2017), and a comparison of proportions of locally differentiated OTUs and/or endemic mycobiont and photobiont species. AR = Argentina, CL = Chile.

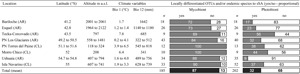

Assignment of unknown accessions to mycobiont species was also based on morphological data, but these data are not included here. Extending the analyses to include morphological characters would increase their complexity and result in an unacceptable decrease in clarity, while also resulting in either the molecular or morphological datasets, or more probably both, not receiving the full treatment they deserve. Therefore, the analysis of the morphological data, along with the taxonomic consequences resulting from the analyses of both datasets, will be dealt with in a separate publication.

#### 

##### Lecidea/Porpidia/Poeltidea–ITS

(Fig. 3, Supplementary Material Files S2-1a & b). This molecular phylogeny includes all relevant taxonomically identified sequences of the genera *Lecidea*, *Porpidia*, *Poeltidea* and *Cyclohymenia* provided by the project framework of the first author or downloaded from the NCBI database (GenBank) to place the newly generated sequences of this study into a global context. Redundant sequences in terms of characters and distribution were not included. The final data matrix for the phylogeny comprised 204 single nrITS sequences with a length of 598 characters and includes sequences of specimens of the genera *Lecidea* (157) *Porpidia* (42), *Poeltidea* (3) and *Cyclohymenia* (2); it was rooted with *Farnoldia jurana* subsp. *jurana* as outgroup. Figure 3 displays the collapsed tree at OTU level, the voucher information included in the OTUs is provided in Supplementary Material File S2-1a and the complete tree is shown in S2-1b. The phylogenetically delimited groups revealed were assigned to OTU-, species-, cluster- and genus level.

All three analyses (distance-based, model-based and bGMYC) showed similar topologies, but at different levels. The groups gained with a distance approach (OTUs) were used to show intraspecific and/or cryptic speciation; model-based approaches were used for the assignment at species level; clusters based on bGMYC were used for grouping closely related species or dividing highly heterogeneous species.

Altogether, 121 OTUs (87 *Lecidea*, 30 *Porpidia*, 1 *Cyclohymenia*, 3 *Poeltidea*) were classified. These OTUs were assigned to species-groups (25 *Lecidea*, 18 *Porpidia*, 1 *Cyclohymenia*, 2 *Poeltidea*) and clusters (19 *Lecidea*, 8 *Porpidia*, 1 *Cyclohymenia*, 3 *Poeltidea*).

The backbone of this phylogeny is not supported but at least four main groups can be recognized (Fig. 3). The first group, with low support, is formed solely by species of the genus *Lecidea* s. str. However, it can be considered a consistent group because it is strongly supported by the three-marker phylogeny (Supplementary Material File S2-2). The Southern Hemisphere lineage (*Porpidia navarina*) is situated outside the *Lecidea* group and next to the second group, which is an intermixed group of *Lecidea* and *Porpidia* species including two well-supported accessions of the genus *Cyclohymenia*. The third group is formed solely by species of the genus *Porpidia* and the fourth by the Southern Hemisphere genus *Poeltidea*.

The *Lecidea* group forms 17 clusters. The well-supported, heterogeneous and cosmopolitan species cluster L01 is formed by the *L. atrobrunnea* (Ramond ex Lam. DC.) Schaer. clade with one southern polar OTU (Lcd05) and a well-supported sister group containing *Lecidea* sp. 1 (Lcd09a, b; endemic to sSA), *L. promiscens* Nyl. (cosmopolitan) including three sSA OTUs (Lcd10-12), and *L. swartzioidea* Nyl. (Northern Hemisphere). Cluster L02 includes *L. confluens* (Weber) Ach. and an accession from GenBank (*Porpidia speirea* (Ach.) Kremp; Schmull *et al*. 2011). The placement of this latter accession in the *Lecidea* s. str. group could be caused by an incorrect species assignment (*Lecidea confluens* and *P. speirea* are morphologically quite similar and mainly distinguished by the ascus-type). This assumption is supported by two accessions of *P. speirea* from China that are included in the phylogeny and are part of cluster P03. This agrees with the findings of Buschbom & Mueller (2004), who described this species as closely related to *P. tuberculosa* (Sm.) Hertel & Knoph, which was confirmed in this phylogeny.

Cluster L03 (*Lecidea* sp. 2) forms a highly supported and very homogenous clade, comprising specimens from Antarctica, the Arctic and sSA, and is formed by a single cosmopolitan OTU (Lcd18).

Cluster L04 is formed by the cosmopolitan *L. andersonii* Filson complex and *Lecidea* sp. 3 clade related to *L. laboriosa* Müll. Arg. (Northern Hemisphere), a Californian specimen of *Lecidea violascens* H. Magn. and an undetermined specimen from the Austrian Alps (*Lecidea* sp. UR00280, Lcd34). The first group contains distinct OTUs of Antarctic, North American and Chinese species of *L. polypycnidophora* U. Rupr. & Türk, the cosmopolitan *L. andersonii*, the Antarctic *Lecidea* sp. UCR1 and two not assignable accessions from North America. The second group (*Lecidea* sp. 3 clade) is formed solely of species from southern South and North America, including OTUs (Lcd27-32) containing specimens from both areas.

Several well-distinguished species form strongly supported clusters, such as the variable accessions from the Northern Hemisphere and maritime Antarctica of the *L. silacea* Ach. clade together with an unidentified specimen from sSA (*Lecidea* sp. UR00130, Lcd 40, cluster L05) and *L. fuscoatrina* Hertel & Leuckert (cluster L06), followed by *L. plana* (J. Lahm) Nyl. (Northern Hemisphere; cluster L07), *L. lithophila* (Ach.) Ach. (Northern Hemisphere, maritime Antarctica; cluster L08), and by the Northern Hemisphere species *L. leucothallina* Arnold, *L. obluridata* Nyl. and an unidentified accession (MK990108, Lcd46) from North America (cluster L09).

Two recently described species, *Lecidea aptrootii* M. Khan *et al*. (Khan *et al.* 2018) and *L. uniformis* McCune (McCune *et al*. 2017), are molecularly very closely related to the accession of *L. fuscoatra* (L.) Ach. var. *grisella* (Flörke) Nyl. (Zhao *et al*. 2016), a taxon which was recognized by Aptroot & Van Herk (2007) at the species level as *L. grisella*. Together with *L. fuscoatra*, they form the strongly supported cluster L10. This is sister to cluster L11, which includes the Antarctic (OTU Lcd58) and, so far, endemic species *L. cancriformis* and closely related, but heterogeneous, accessions solely from sSA (Lcd52-60). Another new and endemic species from sSA (*Lecidea* sp. 4, Lcd61-64) forms cluster L12, and an unidentified specimen (UR00096, Lcd65) forms the highly supported cluster L13.

Cluster L14 is formed by the very common, heterogeneous and cosmopolitan species group *L. lapicida* (Ach.) Ach. It includes the cosmopolitan *L. lapicida* var. *pantherina* (Ach.) Ach. and is sister to the Northern Hemisphere *L. lapicida* var. *lapicida* (Ach.) Ach. These subspecies show no morphological differentiation and are separated only by their different chemotypes but, according to Hertel (1995), they have different ecological requirements. However, cluster L14 contains the two most common OTUs restricted to sSA (Lcd66 and Lcd70), as well as OTUs restricted to Antarctica (Lcd70), the Northern Hemisphere or with an alpine-Antarctic distribution (Lcd67). The now cosmopolitan species *L. medusula* (C. W. Dodge) Hertel, previously reported only for the Southern Hemisphere (Fryday & Hertel 2014), forms cluster L15 with specimens from sSA, the Arctic and the Austrian Alps at the base of this clade. Finally, an Arctic specimen of *L. protabacina* Nyl. and an unidentified specimen from continental Antarctica (T48883b, Lcd80) form the clusters L16 and L17. The species included in this group are all part of *Lecidea* s. str. (Hertel 1984).

The second group, which contains species of the genera *Lecidea* s. str. and *Porpidia*, is highly supported, with the Southern Hemisphere *L. kalbii* Hertel (cluster L19) and the newly described species/genus *Cyclohymenia epilithica* McCune & M. J. Curtis (cluster C01) basal to the group, although with weak support. The cosmopolitan species clade *L. auriculata* Th. Fr. is divided at OTU level into Northern and Southern Hemisphere accessions (Lcd81a,b-84) and *L. tessellata* Flörke is formed by a single cosmopolitan OTU (Lcd85, cluster L18). The well-supported, Northern Hemisphere group of *Porpidia* species includes *P. albocaerulescens* (Wulfen) Hertel & Knoph, *P. degelii* (H. Magn.) Lendemer, *P. rugosa* (Taylor) Coppins & Fryday (cluster P01), *P. hydrophila* (Fr.) Hertel & A. J. Schwab (cluster P02) and *P. flavicunda*, *P. melinodes* (Korb.) Gowan & Ahti, *P. speirea* and *P. tuberculosa* (cluster P03). However, species of the genus *Porpidia* are clearly distinguishable morphologically from species of *Lecidea*, by their ascus type and additionally by the associated green microalgae of the genus *Asterochloris* (Wirth *et al.* 2013; Ruprecht *et al*. 2016).

The third group is formed by four clusters of *Porpidia* species and is divided into two well-supported main subgroups. One is formed by cluster P05, which includes the heterogeneous cosmopolitan clade *inc. sed*. *Porpidia* sp. 1 together with *P. cinereoatra* (Ach.) Hertel & Knoph, *P. contraponenda* (Arnold) Knoph & Hertel, *P. musiva* (Korb.) Hertel & Knoph, *P. irrigua* A. Orange and *Porpidia* sp. (UR00248, Porp17), with *P. striata* Fryday and *P. islandica* Fryday *et al.* forming the highly supported clusters P06 and P07. The other strongly supported subgroup is formed by the heterogeneous and cosmopolitan cluster P08, including *P. flavocruenta* Fryday & Buschbom, several heterogeneous accessions of *P. macrocarpa* (DC.) Hertel & A. J. Schwab, and the quite differing accessions from Turkey and China that were identified as *P. crustulata* (Ach.) Hertel & Knoph.

The genus *Poeltidea*, which occurs only in the Southern Hemisphere, forms the fourth group and consists of three clusters, POE01 and POE02 (*Poeltidea perusta* (Nyl.) Hertel & Hafellner clade), and POE03 (*Poeltidea* sp. 1).

##### Lecidea/Porpidia/Poeltidea–ITS/mtSSU/RPB1

(Supplementary Material File S2-2). The final data matrix of this phylogeny contains 204 concatenated sequences of the markers ITS, mtSSU and *RPB1* with a length of 2115 characters; it includes sequences of specimens of the genera *Lecidea*, *Porpidia* and *Poeltidea* and was rooted with *Farnoldia jurana* subsp. *jurana* as outgroup. Specimens from the project framework of the first author (Antarctica, Arctic, Austria) with the same three markers were added.

All available sequences from this study were included to show the abundance of the different specimens in the studied areas of sSA. The phylogenetically delimited groups revealed were assigned to OTU-, species- and genus level.

This multi-marker phylogeny is not fully comparable to the overall single marker (ITS) phylogeny of *Lecidea*/*Porpidia*/*Poeltidea* because of the limited availability of sequences of the chosen markers in GenBank. The topology is, in most cases, similar and it forms the same four groups, but in many cases they show greater support. The *Lecidea* group, at least, is strongly supported in this phylogeny. Due to the limited availability of *Porpidia* sequences in GenBank, the topology of this group is slightly different, with *P. navarina* clustering together with *P. cinereoatra* and *P. macrocarpa*, but with low support. Finally, the genus *Poeltidea* with two species is still at the base of this phylogeny.

The OTUs obtained from the ITS sequences are still very well supported. Many clades clearly show a local differentiation at OTU level and are endemic to sSA. The two largest clades (OTUs Lcd66 and Lcd70) are part of the cosmopolitan *L. lapicida* cluster and are the most abundant accessions. These are followed by *Porpidia navarina* and *Lecidea* sp. 1 (both species endemic to sSA), *L. promiscens* (cosmopolitan species but with sSA OTUs) and other smaller groups.

##### Lecidella–ITS

(Fig. 4). To place the new accessions of *Lecidella* from sSA into a global context, the species concept and most of the published accessions of Zhao *et al*. (2015) were used. Additionally, several sequences from GenBank and from the project framework of the first author were added to this dataset. Redundant sequences in terms of characters and distribution were not included. The final data matrix of this phylogeny contains 76 sequences of the marker ITS with a length of 538 characters, and was rooted with species of the genera *Carbonea*, *Lecanora* and *Rhizoplaca* to obtain well-defined units in the genus *Lecidella*.

The backbone of the phylogeny is unresolved but four strongly supported main clades are formed; three (*L. enteroleucella* (Nyl.) Hertel, *L. stigmatea* (Ach.) Hertel & Leuckert and *L. elaeochroma* (Ach.) Choisy) are the same as those identified by Zhao *et al*. (2015), with the fourth clade (*Lecidella* spp. nov.) containing two species occurring only in Antarctica and sSA. All available sequences for this group were included to show the abundance of the different accessions. The phylogenetically delimited groups revealed were assigned to OTU-, clade- and genus level.

*Lecidella enteroleucella* is still the only member of the first clade as described by Zhao *et al*. (2015).

The second clade (*L. stigmatea)* is completely unresolved. *Lecidella patavina* (A. Massal.) Knoph & Leuckert and *L. stigmatea* are intermixed and not assignable. These two species differ most noticeably in that the hymenium of *L. patavina* is inspersed with oil droplets, whereas that of *L. stigmatea* is not. That the accessions of these two species are intermixed perhaps indicates that either only one species is involved or that the defining character has been interpreted inconsistently. *Lecidella greenii* U. Rupr. & Türk and *L. siplei* (C. W. Dodge & G. E. Baker) May. Inoue form well-supported lineages and the 12 sequences of the species in this study show a clear local differentiation at OTU level.

A third, new and strongly supported, clade (*Lecidella* spp. nov.) shows two new species from sSA (*Lecidella* sp. 1) and continental Antarctica (*Lecidella* sp. 2).

The fourth clade (*L. elaeochroma*) shows a small number of separated and well-supported species (*L. tumidula* (A. Massal.) Knoph & Leuckert, *L. meiococca* (Nyl.) Leuckert & Hertel, *L. wulfenii* (Hepp) Korb, *L. flavosorediata* (Vězda) Hertel & Leuckert and *Lecidella* sp. 3, which is endemic to sSA). *Lecidella elaeochroma*, *L. euphorea* (Flörke) Hertel, *L. carpathica* Körb., *L. elaeochromoides* (Nyl.) Knoph & Hertel and *L. effugiens* (Nilson) Knoph & Hertel are not assignable because of mingling in different highly supported lineages. None of the investigated specimens were morphologically similar to the Southern Hemisphere species *Lecidella sublapicida* (C. Knight) Hertel (Knoph & Leuckert 1994).

##### Asterochloris–ITS

(Fig. 5). All the *Porpidia* species in this study are not only associated with *Trebouxia* as photobiont, but also with green microalgae of the genus *Asterochloris*. The accessions obtained from sSA were placed into a global context by adding all relevant taxonomically identified sequences from GenBank and from the project framework of the first author. Redundant sequences in terms of characters and distribution were not included. The final data matrix of this phylogeny contains 73 ITS sequences with a length of 519 characters.

The phylogeny was rooted midpoint and divided into two main clades (genera), *Asterochloris* and *Vulcanochloris* Vancurová. The accessions from sSA occur only in the *Asterochloris* clade. The backbone of this clade is unresolved. Again, all available sequences for this group are included to show the abundance of the different accessions. The phylogenetically delimited groups revealed were assigned to OTU-, clade-, and genus level. The topology of the species from GenBank shows a similar pattern to the phylogeny already described in Ruprecht *et al*. (2014).

Only one accession from sSA clusters together with the cosmopolitan species *A. woessiae* Škaloud & Peksa. A highly supported and homogeneous clade is formed by 19 specimens (OTU Ast24) occurring only on Isla Navarino and one accession from the other side of the Beagle Channel (Tierra del Fuego) in the southernmost part of sSA. Four other accessions (OTUs Ast20–23) are placed in the main group with low support.

##### Trebouxia–ITS

(Fig. 6). To place the new accessions of *Trebouxia* from sSA into a global context, the species/OTU concept and the dataset reduced to one accession of each OTU of Leavitt *et al*. (2015) was used. Additionally, several new sequences from GenBank and from the project framework of the first author were added to this dataset. Redundant sequences in terms of characters and distribution were not included. The final data matrix of this phylogeny contains 157 ITS sequences with a length of 805 characters and was midpoint-rooted.

The phylogenetically delimited groups revealed were assigned to OTU- and clade level, as described in Leavitt *et al*. (2015). The backbone of the phylogeny is unresolved but four strongly supported main clades were formed (A, I, S and G) that correspond to those of Leavitt *et al*. (2015).

Altogether, clade A includes 39 OTUs with the specimens of this study forming part of two cosmopolitan (A02 and A04) and four locally differentiated (A36–A39) OTUs. A04 has a sequence similarity of 96.7% and was subdivided into two subunits (A04a and A04b).

Three different groups were formed by accessions assigned to Clade I. Due to a sequence similarity below the threshold of 97.5%, the subOTU I01i of Leavitt *et al.* (2015) was divided into two subunits (I01i and I01j) with a cosmopolitan and southern polar distribution, plus an independent OTU I17 occurring solely in sSA. The addition of the diverse accessions from sSA to the existing OTU I01, which already has 10 subOTUs (I01a–i) described by Leavitt *et al*. (2015), resulted in most of them being transferred into distinct OTUs. However, these have not been renamed here since this is an open system and a regrouping with new accessions is expected in the future.

Fifty percent of the sSA accessions of this marker are contained in the cosmopolitan OTUs S02 and S07 of clade S. S02 was subdivided into four subunits (S02, S02a by Leavitt *et al.* (2015) and S02b, S02c in this study), with the accessions from sSA being assigned only to S02 together with Northern Hemisphere specimens. S02b is formed solely by a specimen from continental Antarctica; a similar finding was described in Ruprecht *et al.* (2012*a*; *T. jamesii* ssp. is equivalent to S02b in this study). Another strongly supported OTU (S16) consists only of accessions from sSA. The cosmopolitan OTU S20 contains three accessions from the southernmost areas of sSA and is part of a strongly supported clade dominated by specimens from Iceland.

No accession from this study is part of clade G.

##### Trebouxia–ITS/psbJ-L/COX2

(Supplementary Material File S2-3). The data matrix of this phylogeny contains 217 concatenated sequences of the markers ITS, psbJ-L and COX2 with a length of 1693 characters. Only sSA specimens are included to demonstrate the intraspecific differentiation. This dataset was calibrated with four cultured *Trebouxia* specimens (see Supplementary Material File S1-3d; 2 × S02/Antarctica, A02/Antarctica and A12/Sweden). Interestingly, the psbJ-L sequences of A02 of the cultured specimen from Antarctica are different from the North American specimen from Leavitt *et al.* (2015).

The dataset still shows the same number of OTUs as the ITS phylogeny (Fig. 6) and the grouping is the same. Clade A shows nine well-supported groups at species level (A02, A12, A38, A39, A04a, A04b, A36, A37), with A36 and A04b being closely related. The subOTUs A04a and A04b are more separated than in the ITS phylogeny due to the marker psbJ-L.

Clade I is divided into two subOTUs (I01i and I01j) and one newly developed distinct OTU (I17) because of its heterogeneous structure. No COX2 sequences were included because they were not assignable.

More than half of the sequences are included in the homogenous and cosmopolitan OTUs S02 and (a smaller part) in S07. These groups are closely related and share similar COX2 sequences. OTU S16, which occurs only in sSA, is clearly separated at species level from S02 and S07, and S20 is situated at the base of clade S.

### Distribution of species and OTUs: globally distributed vs. restricted to sSA and/or southern polar regions

Altogether, 185 mycobiont specimens forming 54 OTUs assigned to 24 species of the genera *Lecidea*, *Porpidia*, *Poeltidea* and *Lecidella* were identified. Four species of the genus *Lecidea* (*Lecidea* sp. 2, *Lecidea* sp. 3, *L. medusula*, *L. tessellata*) and *Porpidia macrocarpa* are globally distributed at species and OTU level.

By far the most abundant accessions are formed by locally differentiated OTUs (Lcd66, Lcd70; 48 accessions) and belong to the cosmopolitan species-clade *Lecidea lapicida* (cluster L14), with the next most frequent groups being *Lecidella stigmatea* (13) and *Lecidea promiscens* (9). Finally, a total of 12 species or single sequences are so far described and/or known only from sSA or the southern polar regions (Table 1, Figs 3 & 4, Supplementary Material Files S2-1a, b & S2-2).

The photobionts comprise 202 accessions that are assigned to 20 OTUs. Most of the algal specimens (128) belong to globally distributed taxa, especially Tr_S02 with 84 accessions, followed by Tr_S07, Tr_A02, Tr_A12, Tr_A04a, Tr_S20 and a single accession of *Asterochloris woessiae* from a lower elevation area close to Esquel, Argentina. The very heterogeneous OTU Tr_I01i (Leavitt *et al.* 2015) was divided into two subOTUs: I01i with a global distribution and I01j that occurs in Antarctica and sSA. OTU I17 occurs solely in sSA. A surprisingly high number of 74 accessions (*Asterochloris* and *Trebouxia*) form 13 clearly separated OTUs and have, so far, been found only in sSA (Table 2, Figs 5 & 6, Supplementary Material File S2-3).

In summary, for the mycobiont the percentage distribution of accessions at OTU level shows a high rate of local differentiation and endemism for the sSA specimens versus those that are globally distributed (**87**:13). In particular, Parque Nacional Torres del Paine and Morro Chico, with 100% each, and the southernmost sampling point, Isla Navarino, with 90% have the highest amount of specialized accessions. In contrast, the photobiont OTUs show a higher rate of globally distributed accessions (32:**68**). However, both symbionts show no significant specialization along the latitudinal gradient in southern South America (Table 3).

## Discussion

For the four re-evaluated groups *Lecidea*/*Porpidia*/*Poeltidea*, *Lecidella* (mycobiont), *Asterochloris* and *Trebouxia* (photobiont), the geographically isolated southern end of the South American continent supports a high degree of locally differentiated subclades (OTUs) in globally distributed species, as well as lineages currently known only from sSA at the species, coalescent-defined cluster and genus level. This was, to some extent, unexpected for these mostly globally distributed genera and can partially be explained by the lack of sequence information for most of the Southern Hemisphere lecideoid mycobiont species (Knoph & Leuckert 1994; Fryday & Hertel 2014). This also applies to the photobionts because of the limited availability of molecular studies for the southern polar regions (e.g. Muggia *et al*. 2010; Fernández-Mendoza *et al*. 2011; Ruprecht *et al*. 2012*a*).

Local differentiation (cryptic speciation) appears quite common in lichen-forming fungi (Leavitt *et al.* 2011; Lumbsch & Leavitt 2011; Kraichak *et al*. 2015; Dal Grande *et al*. 2017), but has rarely been described for the most common and widespread lichen photobiont taxa *Trebouxia* (Fernández-Mendoza *et al*. 2011; Ruprecht *et al*. 2012*a*; Leavitt *et al.* 2015) and *Asterochloris* (Škaloud *et al*. 2015).

In particular, the lichen cluster (L14, Fig. 3) *Lecidea lapicida*, which occurs in polar and high mountainous regions worldwide (Hertel 1984; Hertel & Andreev 2003; Hafellner & Türk 2016), shows locally differentiated accessions at the OTU level that are currently known only from sSA. Surprisingly, Austrian and Antarctic (Lcd67) accessions are closely related, but they are clearly distinct from the two main OTUs (Lcd66 and Lcd70) that comprise 48 of the 185 sSA specimens. Another cosmopolitan (Northern Hemisphere and sSA) and common lichen species is *L. auriculata*, which includes a quite homogenous but widely distributed OTU (Lcd81b, 13 specimens) restricted to sSA. The heterogeneous clades of *Porpidia macrocarpa*, which include specimens from Antarctica and the Austrian Alps, show a similar pattern. *Lecidea atrobrunnea* is the only exception, forming two southern polar OTUs with accessions from both Antarctica and sSA (Fig. 3). A similar pattern is also known for *Usnea aurantiacoatra* (Jacq.) Bory (Laguna Defior 2016) and *Cetraria aculeata* (Fernández-Mendoza *et al*. 2011). A different example is *Lecidea cancriformis*, which has, so far, been described as endemic to Antarctica and is one of the dominant crustose lichens in the most extreme areas of the continent (Castello 2003; Hertel 2007; Ruprecht *et al*. 2010). The Antarctic accessions belong to a single, well-supported OTU (Lcd58; Fig. 3, Supplementary Material Files S2-1a, b & S2-2, available online) but there are seven closely related OTUs occurring in sampling areas north of Tierra del Fuego. However, the whole cluster L11, including *L. cancriformis*, remains confined to the southern polar regions. Several other species, as well as the genus *Poeltidea*, occur solely in the southern polar regions. *Lecidea medusula*, which was previously only investigated morphologically and thought to be endemic to the Southern Hemisphere (Hertel 2009), is shown to be a cosmopolitan species. However, in total, the cosmopolitan species/OTUs (*Lecidea* sp.2, *Lecidea* sp.3, *L. medusula*, *L. tessellata*, *Porpidia macrocarpa*) are outnumbered by those that are currently known only from sSA (Table 1).

The *Lecidella* phylogeny, based on the data of Zhao *et al*. (2015), reveals a new southern polar species-level clade (*Lecidella* spp. nov.) with accessions from sSA and continental Antarctica. The specimens are not morphologically assignable to the available species descriptions (e.g. Knoph & Leuckert 1994; Ruprecht *et al*. 2012*b*; Wirth *et al*. 2013). All the other sequences added to the phylogeny of Zhao *et al*. (2015) form well-supported and distinguished OTUs endemic to sSA.

Interestingly, several cosmopolitan and abundant mycobiont species occurring in continental and maritime Antarctica, for example *Lecidea andersonii*, *L. polypycnidophora* (Hertel 2007; Ruprecht *et al*. 2010, 2016; Hale *et al*. 2019) and *Lecidella siplei* (Ruprecht *et al*. 2012*b*), were not found in the sSA regions.

In contrast to the mycobiont, the photobiont shows the opposite pattern. In particular, the genus *Trebouxia* is known as widely distributed globally, with often low diversification (Muggia *et al*. 2010; Ruprecht *et al.* 2012*a*). These findings are supported in this study with more than half of the accessions assigned to two cosmopolitan OTUs of the genus *Trebouxia* (S02 and S07) plus some smaller groups (A02, A04a, A12; Leavitt *et al*. 2015; Fig. 6, Table 2, Supplementary Material File S2-3). The remaining *Trebouxia* and *Asterochloris* accessions form highly diverse and locally differentiated and/or endemic groups, which was unexpected. The contrasting distribution behaviour of the cosmopolitan photobionts could be caused mainly by their wide choice of mycobiont partners (Kroken & Taylor 2000), allowing them access to the different distribution strategies of the various lichens.

The most southern sampling area at Isla Navarino shows, for the mycobiont, not only locally restricted OTUs, but also strongly supported endemic species (e.g. *Porpidia navarina*; Ruprecht *et al*. 2016), which is also the case for the photobiont *Asterochloris* (OTU Ast24). As this area was ice-free during the Last Glacial Maximum (Douglass *et al*. 2005), the likely reason can be explained by the concept of glacial refugia, where the cold and glacial phases were the drivers of population divergences and (cryptic) speciation after transition from the Northern to the Southern Hemisphere (Paula & Leonardo 2006; Stewart *et al*. 2010; Fernández-Mendoza & Printzen 2013). Furthermore, the two most southern areas of the South American continent sampled (Parque Nacional Torres del Paine and Morro Chico), which both have 100% locally differentiated OTUs and endemic species for the mycobiont (Table 3), are influenced by the violent westerly gales caused by the split of the Humboldt Current to the north and the Antarctic Circumpolar Current (ACC) to the south (Silva *et al.* 2009). This further leads to the assumption that mycobiont dispersal is limited through this asymmetrical wind system, which is caused by the undertow of the ACC, driven by westerly winds over the circumpolar streamlines (Allison *et al*. 2010). Moreover the flow speed of the ACC between the Last Glacial Maximum and the Holocene has remained almost unchanged (McCave *et al.* 2014). Nonetheless, several other strongly supported species groups that occur only in other areas of sSA, such as *Lecidella* sp. 1 at 2000 m a.s.l. close to Esquel (42.8°S) or endemic lineages in *Trebouxia* (S16, A38, A39), hint at further, and so far unknown, separation events at the remote and climatically extreme southern end of the American continent.

### Taxonomy

It is well known that the mycobiont genera *Lecidea* and *Porpidia* (Fig. 3) are not clearly separated and our phylogeny confirms that the species currently included in *Porpidia* do not form a monophyletic group (i.e. Buschbom & Mueller 2004; Schmull *et al.* 2011; Ruprecht *et al.* 2016; Fig. 3, Supplementary Material Files S2-1 & S2-2b). Additionally, the newly described species/genus *Cyclohymenia epilithica* with perithecioid apothecia and an apparently *Porpidia*-type ascus (McCune *et al.* 2017) is situated among these two genera. In general, species of *Lecidea* and *Porpidia* are morphologically differentiated by ascus-type (*Lecidea* or *Porpidia*), larger ascospores with the presence of a perispore in *Porpidia* and different genera of associated green microalgae as photobionts (*Trebouxia* sp. in *Lecidea* sp., *Asterochloris* sp. and *Trebouxia* sp. in *Porpidia* sp.; Ruprecht *et al.* 2016). *Chlorella* sp. as described by Li *et al.* (2013) was not found in the sSA *Porpidia* species. Although several new sequences were added for species that were previously only described morphologically (e.g. *L. kalbii*, *L. promiscens*, *L. swartzioidea, L. lithophila*, *Poeltidea perusta*), and sequences for other *Porpidia* species obtained from GenBank, the re-evaluated phylogeny could not be resolved. However, three species morphologically assigned to *Lecidea* s. str. (*L. auriculata, L. tessellata*, *L. kalbii*; Hertel 1984; Wirth *et al.* 2013; Fryday & Hertel 2014) form, together with several *Porpidia* species and the genus *Cyclohymenia*, a highly supported but intermixed group. Although clearly defined groups for *Porpidia* are easily recognized and a name at the genus level is available for at least one of them (*Haplocarpon*), it would be premature to formally recognize these groups as genera because of the uncertain systematic position of the rare type species, *Porpidia trullisata* (Kremp.) Körb, for which molecular data are not yet available. However, the three other groups in our phylogeny are formed solely by species of *Lecidea* s. str., *Porpidia* and *Poeltidea*, respectively.

The unresolved and intermixed topology of several species in the two main clades of the *Lecidella* phylogeny (*L. stigmatea* and *L. elaeochroma*; Fig. 4) could not be improved with the additional specimens from sSA. Only an extended species sampling can help to unravel the inconsistent relationships in both these phylogenies (*Lecidea*/*Porpidia*/*Poeltidea*, Fig. 3; *Lecidella*, Fig. 4).

## Conclusions

The species-rich group of lecideoid lichens found extensively in alpine and polar regions in southern South America comprises highly divergent OTUs of cosmopolitan species, as well as several endemic species. Three factors may contribute to the observed differentiation and endemism: a) the geographical isolation of this southernmost landmass north of Antarctica, b) limited dispersal caused by the Antarctic Circumpolar Current system, and c) the presence of regional glacial refugia.

The diverging patterns of dispersal in the cosmopolitan lecideoid lichen group are still under-researched. Acquiring larger datasets along the assumed distribution routes of the highest mountain ranges (Garrido-Benavent & Pérez-Ortega 2017; Hale *et al*. 2019), and a consequent sampling for better global coverage, will help to understand colonization events and specialization in this, so far, quite overlooked group of crustose lichens.

## Supplementary material

For supplementary material accompanying this paper visit http://dx.doi.org/10.1017/S0024282920000225. click here to view supplementary material
