# Supplementary material for: High levels of endemism and local differentiation in the fungal and algal symbionts of saxicolous lecideoid lichens along a latitudinal gradient in southern South America
Source: Lichenologist (Lond). 2020 Jul 29;52(4):287–303. doi: 10.1017/S0024282920000225 (PMC7396322; doi:10.1017/S0024282920000225)
Supplement: Supplementary file 1 [file S0024282920000225sup001.zip › S0024282920000225sup001/S1_200520.pdf]

Supplementary Material S1

Table of Contents

S1-1…………………….. 1 - 3
S1-2a…………………… 1 - 6
S1-2b…………………… 6 - 8
S1-2c…………………… 9 - 13
S1-2d…………………… 13
S1-3……………………. 1 - 2
S1-4……………………. 1
S1-5……………………. 1

S1-1 Investigation sites: location_id, location (country, region, area), altitude, latitude, longitude, CHELSA climate variables BIO1 (annual mean temperature) and BIO12 (annual precipitation; Karger *et al.* 2017).

**location_id**	**country**	**region**	**area**	**altitude** m a.s.l.	**latitude**	**longitude**	**bio1**	**bio12**
TdF_GM_1	Argentina	Tierra del Fuego, Ushuaia	Glaciar Martial	570	-54,78991798	-68,39034302	1,6	489
TdF_GM_2	Argentina	Tierra del Fuego, Ushuaia	Glaciar Martial	587	-54,78952697	-68,39129596	1,6	489
TdF_GM_3	Argentina	Tierra del Fuego, Ushuaia	Glaciar Martial	593	-54,789	-68,39313998	1,8	526
TdF_CB_1	Chile	Tierra del Fuego, Región de Magallanes y de la Antártica Chilena, Isla Navarino	Cerro Bandera	607	-54,96288098	-67,634564	2,7	701
TdF_CB_2	Chile	Tierra del Fuego, Región de Magallanes y de la Antártica Chilena, Isla Navarino	Cerro Bandera	623	-54,96410097	-67,63851698	2,7	701
TdF_CB_3	Chile	Tierra del Fuego, Región de Magallanes y de la Antártica Chilena, Isla Navarino	Cerro Bandera	619	-54,96530604	-67,64167897	2,7	701
TdF_CB_4	Chile	Tierra del Fuego, Región de Magallanes y de la Antártica Chilena, Isla Navarino	Cerro Bandera	657	-54,97040299	-67,64538897	2,3	737
TdF_CB_5	Chile	Tierra del Fuego, Región de Magallanes y de la Antártica Chilena, Isla Navarino	Cerro Bandera	694	-54,973166	-67,642288	2,3	737
TdF_CB_6	Chile	Tierra del Fuego, Región de Magallanes y de la Antártica Chilena, Isla Navarino	Cerro Bandera	702	-54,97335803	-67,64681004	2,3	737
TdF_CB_7	Chile	Tierra del Fuego, Región de Magallanes y de la Antártica Chilena, Isla Navarino	Cerro Bandera	688	-54,97366698	-67,65027696	3,3	650
TdF_CB_8	Chile	Tierra del Fuego, Región de Magallanes y de la Antártica Chilena, Isla Navarino	Cerro Bandera	680	-54,97164301	-67,632565	2,6	628
TdF_CB_9	Chile	Tierra del Fuego, Región de Magallanes y de la Antártica Chilena, Isla Navarino	Cerro Bandera	730	-54,97210502	-67,63245997	2,6	628
TdF_CB_10	Chile	Tierra del Fuego, Región de Magallanes y de la Antártica Chilena, Isla Navarino	Cerro Bandera	744	-54,97210301	-67,63207901	2,6	628
TdF_CB_11	Chile	Tierra del Fuego, Región de Magallanes y de la Antártica Chilena, Isla Navarino	Cerro Bandera	741	-54,97349499	-67,63205403	2,6	628

**location_id**	**country**	**region**	**area**	**altitude** m a.s.l.	**latitude**	**longitude**	**bio1**	**bio12**
TdF_CB_12	Chile	Tierra del Fuego, Región de Magallanes y de la Antártica Chilena, Isla Navarino	Cerro Bandera	765	-54,97333699	-67,63534997	1,9	739
TdF_CB_13	Chile	Tierra del Fuego, Región de Magallanes y de la Antártica Chilena, Isla Navarino	Cerro Bandera	741	-54,97261003	-67,63830702	1,9	739
TdF_CC_1	Argentina	Tierra del Fuego, Ushuaia	Cerro Castor	683	-54,71255503	-68,00119598	2,2	652
TdF_CC_2	Argentina	Tierra del Fuego, Ushuaia	Cerro Castor	682	-54,712507	-67,99986896	2	648
TdF_CC_3	Argentina	Tierra del Fuego, Ushuaia	Cerro Castor	684	-54,71230902	-67,99831504	2	648
TdF_CC_4	Argentina	Tierra del Fuego, Ushuaia	Cerro Castor	686	-54,71235797	-67,997316	2	648
TdF_CC_5	Argentina	Tierra del Fuego, Ushuaia	Cerro Castor	690	-54,71236903	-67,99557298	2	648
TdF_CC_6	Argentina	Tierra del Fuego, Ushuaia	Cerro Castor	794	-54,71009804	-67,99191898	2	648
TdF_VL_1	Argentina	Tierra del Fuego, Ushuaia	Valle de Lobos	365	-54,69848701	-68,13101297	3,7	448
TdF_VL_2	Argentina	Tierra del Fuego, Ushuaia	Valle de Lobos	369	-54,69806196	-68,130846	3,7	448
TdF_TdF_1	Argentina	Tierra del Fuego, Ushuaia	Parque National de T.d. F.	675	-54,81220898	-68,53777701	2,1	756
TdF_TdF_2	Argentina	Tierra del Fuego, Ushuaia	Parque National de T.d. F.	628	-54,81220697	-68,53777399	2,1	756
TdF_TdF_3	Argentina	Tierra del Fuego, Ushuaia	Parque National de T.d. F.	407	-54,81801404	-68,55260703	4	715
RM_MC_1	Chile	Región de Magallanes y de la Antártica Chilena, Laguna Blanca	Morro Chico	208	-52,05869998	-71,41999399	6,4	341
RM_TdP_1	Chile	Región de Magallanes y de la Antártica Chilena, Patagonia chilena	south of P.N. Torres del Paine	143	-51,57855699	-72,60027903	6,5	818
RM_TdP_2	Chile	Región de Magallanes y de la Antártica Chilena, Patagonia chilena	south of P.N. Torres del Paine	118	-51,26429002	-72,86597597	6,4	545
RM_TdP_3	Chile	Región de Magallanes y de la Antártica Chilena, Patagonia chilena	NP Torres del Paine	135	-51,12115997	-73,14255497	5,4	730
RM_TdP_4	Chile	Región de Magallanes y de la Antártica Chilena, Patagonia chilena	NP Torres del Paine	239	-51,123999	-73,142545	5,4	730
RM_TdP_5	Chile	Región de Magallanes y de la Antártica Chilena, Patagonia chilena	NP Torres del Paine	342	-51,12560699	-73,14431098	3,9	770
SC_CC_1	Argentina	Provinzia de Santa Cruz, El Calaftae	Cerro Cristal	955	-50,55998401	-72,79423401	2	512
SC_CC_2	Argentina	Provinzia de Santa Cruz, El Calaftae	Cerro Cristal	1054	-50,56179198	-72,78976704	1,3	512
SC_CC_3	Argentina	Provinzia de Santa Cruz, El Calaftae	Cerro Cristal	1086	-50,564484	-72,78745397	1,3	512
SC_CC_4	Argentina	Provinzia de Santa Cruz, El Calaftae	Cerro Cristal	1253	-50,56685499	-72,78100502	0,8	492
SC_CC_5	Argentina	Provinzia de Santa Cruz, El Calaftae	Cerro Cristal	994	-50,56108003	-72,79271403	2	512
SC_LP_1	Argentina	Provinzia de Santa Cruz, El Chalten	Lomo del Plague Tumbado	1109	-49,36369599	-72,94175098	1,5	467
SC_LP_2	Argentina	Provinzia de Santa Cruz, El Chalten	Lomo del Plague Tumbado	1163	-49,35924604	-72,94870402	0,9	501
SC_LP_3	Argentina	Provinzia de Santa Cruz, El Chalten	Lomo del Plague Tumbado	1280	-49,35681302	-72,96007801	0,7	585

**location_id**	**country**	**region**	**area**	**altitude** m a.s.l.	**latitude**	**longitude**	**bio1**	**bio12**
SC_LP_4	Argentina	Provinzia de Santa Cruz, El Chalten	Lomo del Plague Tumbado	1388	-49,35929197	-72,96415002	0,2	502
SC_LP_5	Argentina	Provinzia de Santa Cruz, El Chalten	Lomo del Plague Tumbado	1452	-49,35928803	-72,96619101	0,2	502
SC_LP_6	Argentina	Provinzia de Santa Cruz, El Chalten	Lomo del Plague Tumbado	1483	-49,359283	-72,96717203	0	473
SC_LT_1	Argentina	Provinzia de Santa Cruz, El Chalten	path to Laguna de los Tres	558	-49,315723	-72,900999	4,1	322
SC_LT_2	Argentina	Provinzia de Santa Cruz, El Chalten	path to Laguna de los Tres	578	-49,31370498	-72,90347401	4,1	322
SC_LT_3	Argentina	Provinzia de Santa Cruz, El Chalten	path to Laguna de los Tres	705	-49,30902997	-72,91458598	3,4	346
SC_LT_4	Argentina	Provinzia de Santa Cruz, El Chalten	path to Laguna de los Tres	721	-49,29002903	-72,94508597	3,2	392
SC_LT_5	Argentina	Provinzia de Santa Cruz, El Chalten	path to Laguna de los Tres	864	-49,280158	-72,97340297	2,8	368
SC_LT_6	Argentina	Provinzia de Santa Cruz, El Chalten	path to Laguna de los Tres	1103	-49,280762	-72,98022601	1,4	389
SC_LT_7	Argentina	Provinzia de Santa Cruz, El Chalten	Laguna de los Tres	1182	-49,27974704	-72,98398999	0,9	399
CH_TC_1	Argentina	Provincia de Chubhut, Tecka-Corcovado		797	-43,49470097	-71,28400097	7,8	685
CH_AL_1	Argentina	Provincia de Chubhut, Esquel	P.N. de Alerces	882	-42,90906301	-71,63743003	6,9	1143
CH_LH_1	Argentina	Provincia de Chubhut, Esquel	La Hoya	1964	-42,811456	-71,25711199	1,4	1140
CH_LH_2	Argentina	Provincia de Chubhut, Esquel	La Hoya	2011	-42,81073096	-71,25773703	1,4	1140
CH_LH_3	Argentina	Provincia de Chubhut, Esquel	La Hoya	2089	-42,809372	-71,25885802	1,2	1161
CH_LH_4	Argentina	Provincia de Chubhut, Esquel	La Hoya	2109	-42,80920302	-71,259703	1,2	1161
CH_LH_5	Argentina	Provincia de Chubhut, Esquel	La Hoya	2122	-42,80799503	-71,25616098	1,2	1180
CH_CC_1	Argentina	Provincia de Chubhut, Esquel	Cerro La Cruz	1046	-42,9288889	-71,3016139	7,3	985
RN_CC_1	Argentina	Provincia de Rio Negro, Bariloche	Cerro Catedral	2001	-41,17281304	-71,48415403	1,7	1642
RN_CC_2	Argentina	Provincia de Rio Negro, Bariloche	Cerro Catedral	2075	-41,17067096	-71,48645704	1,7	1642
RN_CC_3	Argentina	Provincia de Rio Negro, Bariloche	Cerro Catedral	2061	-41,16764098	-71,48550603	1,7	1642
RN_CC_4	Argentina	Provincia de Rio Negro, Bariloche	Cerro Catedral	2048	-41,17334202	-71,48629401	1,7	1642

References

Karger, D.N., Conrad, O., Bohner, J., Kawohl, T., Kreft, H., Soria-Auza, R.W., Zimmermann, N.E., Linder, H.P. & Kessler, M. (2017) Data Descriptor: Climatologies at high resolution for the earth's land surface areas. *Scientific Data* **4**.

S1-2a Saxicolous lecideoid lichen specimens from southern South America (sSA) collected by the first author and used in this study. Taxonomic information was taken from the following literature: Ruprecht *et al.* (2010, 2016); Fryday & Hertel (2014); Hafellner & Türk (2016).

		**Mycobiont**						**Associated green micro algae**						
**(SZU)**					**Accession numbers**			***Trebouxia***	**Accession numbers**			***Asterochloris* & *Chloroidium***		**Acc. num.**
**Voucher_ID**	**Location_id**	**Species**	**OTU_id**	**Cluster**	**ITS**	**mtSSU**	**RPB1**	**OTU_id**	**ITS**	**psbJ-L**	**COX2**	**Species_name**	**OTU_id**	**ITS**
UR00001	TdF_GM_1	*Lecidea atrobrunnea* (Ramond ex Lam. DC.) Schaer.	LP_Lcd05	L01	MK620100	MK684579	MK684920	Tr_S02	MK619907	MK684744	MK684487			
UR00002	TdF_GM_1	*Lecidea lapicida* (Ach.) Ach.	LP_Lcd70	L14	KX120200	MK684580	MK684921	Tr_S02	MK619908	MK684745	MK684488			
UR00004	TdF_GM_2	*Poeltidea* sp. 1	LP_Poe03	POE03	MK620101	MK684581		Tr_S02	MK619909					
UR00005	TdF_GM_2	*Lecidea cancriformis* (C.W. Dodge & G.E. Baker)	LP_Lcd51	L14	MK659868	MK684582	MK684922	Tr_S02	MK619910	MK684746				
UR00006	TdF_GM_2	*Lecidea medusula* (C.W. Dodge) Hertel	LP_Lcd78	L15	KX120204	MK684583	MK684923	Tr_S02	MK619911	MK684747	MK684489			
UR00008	TdF_GM_3	*Lecidea lapicida* (Ach.) Ach.	LP_Lcd66	L14	KX120202	MK684584	MK684924	Tr_S02	MK619912	MK684748	MK684490			
UR00009	TdF_GM_3	*Lecidea lapicida* (Ach.) Ach.	LP_Lcd66	L14	MK620103	MK684585	MK684925	Tr_S02	MK619913	MK684749	MK684491			
UR00010	TdF_GM_3	*Lecidea auriculata* Th.Fr.	LP_Lcd81bb	L18	KX120212	MK684586	MK684926	Tr_S02	MK619914	MK684750	MK684492			
UR00012	TdF_CB_1	inc. sed. *Porpidia* sp. 1	LP_Porp12	P05	KX120227	MK684587	MK685048	Tr_S02	MK619915	MK684751	MK684493	*A.* sp. URa18	Ast24	KX120177
UR00013	TdF_CB_1	*Porpidia navarina* U. Rupr. & Türk	LP_Porp09	P04	KX120214	MK684588	MK685038	Tr_S02	KX120242	MK684752		*A.* sp. URa19	Ast23	KX120198
UR00014	TdF_CB_1	inc. sed. *Porpidia* sp. 1	LP_Porp11	P05	KX120229	MK684589	MK685049	Tr_S02	KX120245	MK684753		*A.* sp. URa18	Ast24	KX120183
UR00015	TdF_CB_1	*Porpidia navarina* U. Rupr. & Türk	LP_Porp09	P04	KX120215	MK684590	MK685039	Tr_S02	KX120243	MK684754	MK684494	*A.* sp. URa18	Ast24	KX120184
UR00016	TdF_CB_2	inc. sed. *Porpidia* sp. 1	LP_Porp11	P05	KX120230	MK684591	MK685050	Tr_S20	MK619916	MK684755		*A.* sp. URa18	Ast24	KX120184
UR00017	TdF_CB_3	*Lecidea lapicida* (Ach.) Ach..	LP_Lcd70	L14	KX120201	MK684592	MK684927	Tr_S02	MK619917	MK684756	MK684495			
UR00018	TdF_CB_4	*Porpidia navarina* U. Rupr. & Türk	LP_Porp09	P04	KX120216	MK684593	MK685040	Tr_S20	KX120253			*A.* sp. URa18	Ast24	KX120193
UR00020	TdF_CB_5	*Porpidia navarina* U. Rupr. & Türk	LP_Porp09	P04	KX120217	MK684594	MK685041	Tr_A02	KX120249			*A.* sp. URa18	Ast24	KX120178
UR00022	TdF_CB_5	*Porpidia macrocarpa* (DC.) Hertel & A.J.Schwab	LP_Porp20	P08	KX120232	MK684595	MK685051	Tr_A02	KX120247			*A.* sp. URa18	Ast24	KX120191
UR00023	TdF_CB_6	*Porpidia navarina* U. Rupr. & Türk	LP_Porp09	P04	KX120226	MK684596	MK685042	Tr_A04a	KX120251	MK684757	MK684496	*A.* sp. URa18	Ast24	KX120189
UR00024	TdF_CB_7	*Lecidea lapicida* (Ach.) Ach.	LP_Lcd70	L14	MK620104	MK684597	MK684928	Tr_S02	MK619918	MK684758	MK684497			
UR00025	TdF_CB_7	*Lecidea* sp. 4	LP_Lcd61	L12	MK620105	MK684598	MK684929	Tr_A04a	MK619919	MK684759				
UR00026	TdF_CB_7	*Poeltidea* cf. *perusta* (Nyl.) Hertel & Hafellner	LP_Poe01	POE01	MK620106	MK684599	MK684886	Tr_S02	MK619920	MK684760	MK684498			
UR00027	TdF_CB_7	*Porpidia macrocarpa* (DC.) Hertel & A.J.Schwab	LP_Porp20	P08	KX120234	MK684600	MK685052	Tr_A02	KX120248			*A.* sp. UR00027	Ast22	KX120195
UR00029	TdF_CB_7	inc. sed. *Porpidia* sp. 1	LP_Porp12	P05	KX120228	MK684601	MK685053	Tr_S02	MK619921	MK684761		*A.* sp. URa18	Ast24	Kx120190
UR00030	TdF_CB_7	*Porpidia navarina* U. Rupr. & Türk	LP_Porp09	P04	KX120218	MK684602		Tr_A12	KX120252			*A.* sp. URa18	Ast24	KX120185
UR00031	TdF_CB_7	*Porpidia navarina* U. Rupr. & Türk	LP_Porp09	P04	KX120219	MK684603		Tr_S02	MK619922	MK684762		*A.* sp. URa18	Ast24	KX120179
UR00032	TdF_CB_8	*Porpidia macrocarpa* (DC.) Hertel & A.J.Schwab	LP_Porp20	P08	KX120233	MK684604		Tr_S02	MK619923	MK684763		*A.* sp. URa18	Ast24	KX120188
UR00033	TdF_CB_8	*Lecidea lapicida* (Ach.) Ach.	LP_Lcd70	L14	MK620107	MK684605	MK684930	Tr_S02	MK619924	MK684764	MK684499			
UR00034	TdF_CB_9	*Lecidea lapicida* (Ach.) Ach.	LP_Lcd70	L14	MK620108	MK684606	MK684931	Tr_S02	MK619925	MK684765	MK684500			

Voucher_ID	Location_id	Species	OTU_id	Cluster	ITS	mtSSU	RPB1	OTU_id	ITS	psbJ-L	COX2	Species	OTU_id	ITS
UR00035	TdF_CB_10	*Lecidella stigmatea* (Ach.) Hertel & Leuckert	LL_ST15		MK620109			Tr_A12	MK619926	MK684766	MK684501			
UR00036	TdF_CB_11	*Lecidea lapicida* (Ach.) Ach.	LP_Lcd70	L14	MK620110	MK684607	MK684932	Tr_S02	MK619927	MK684767	MK684502			
UR00037	TdF_CB_11	*Poeltidea perusta* (Nyl.) Hertel & Hafellner	LP_Poe02	POE02	MK620111	MK684608	MK684887	Tr_S02	MK619928	MK684768	MK684503			
UR00039	TdF_CB_11	*Poeltidea perusta* (Nyl.) Hertel & Hafellner	LP_Poe02	POE02	MK620112	MK684609	MK684888	Tr_S02	MK619929	MK684769	MK684504			
UR00040	TdF_CB_12	*Lecidea lapicida* (Ach.) Ach.	LP_Lcd70	L14	MK620113	MK684610	MK684933	Tr_S02	MK619930	MK684770				
UR00041	TdF_CB_12	*Lecidea lapicida* (Ach.) Ach.	LP_Lcd70	L14	MK620114	MK684611	MK684934	Tr_S02	MK619931	MK684771	MK684505			
UR00043	TdF_CB_12	*Porpidia navarina* U. Rupr. & Türk	LP_Porp09	P04	KX120220	MK684612	MK685043	Tr_S02	KX120246	MK684772	MK684506	*A.* sp. URa18	Ast24	KX120186
UR00044	TdF_CB_12	*Porpidia navarina* U. Rupr. & Türk	LP_Porp09	P04	KX120221	MK684613	MK685044	Tr_S02	MK619932			*A.* sp. URa18	Ast24	KX120180
UR00045	TdF_CB_13	*Lecidea* sp. 4	LP_Lcd53	L12	MK620115	MK684614	MK684935	Tr_I01i	MK619933					
UR00046	TdF_CB_13	*Porpidia navarina* U. Rupr. & Türk	LP_Porp09	P04	KX120222	MK684615	MK685045	Tr_I01j	KX120238			*A.* sp. URa18	Ast24	KX120194
UR00047	TdF_CB_13	*Porpidia navarina* U. Rupr. & Türk	LP_Porp09	P04	KX120223	MK684616		Tr_I01j	KX120239	MK684773		*A.* sp. URa18	Ast24	KX120181
UR00048	TdF_CB_13	*Porpidia navarina* U. Rupr. & Türk	LP_Porp09	P04	KX120224	MK684617	MK685046	Tr_S02	KX120244	MK684774	MK684507	*A.* sp. URa18	Ast24	KX120192
UR00050	TdF_CB_13	*Lecidea lapicida* (Ach.) Ach.	LP_Lcd70	L14	MK620116	MK684618	MK684936	Tr_I01i	MK619934					
UR00051	TdF_CC_1	*Lecidea lapicida* (Ach.) Ach.	LP_Lcd70	L14	MK620117	MK684619	MK684937	Tr_S02	MK619935	MK684775	MK684508			
UR00052	TdF_CC_1	inc. sed. *Porpidia* sp. 1	LP_Porp11	P05	KX120231	MK684620	MK685054	Tr_S02	KX120240	MK684776	MK684509	*A.* sp. URa18	Ast24	KX120182
UR00055	TdF_CC_1	*Lecidea lapicida* (Ach.) Ach.	LP_Lcd70	L14	MK620118	MK684621	MK684938	Tr_S02	MK619936					
UR00056	TdF_CC_1	*Porpidia navarina* U. Rupr. & Türk	LP_Porp09	P04	KX120225	MK684622	MK685047	Tr_S20	KX120254		MK684510	*A.* sp. URa18	Ast24	KX120187
UR00057	TdF_CC_2	*Poeltidea* sp. 1	LP_Poe03	POE03	MK620119	MK684623						Chl. sp. UR00057		MK620073
UR00058	TdF_CC_2	*Poeltidea* sp. 1	LP_Poe03	POE03	MK620120	MK684624		Tr_S02		MK684777		Chl. sp. UR00058		MK620074
UR00059	TdF_CC_3	*Lecidea lapicida* (Ach.) Ach.	LP_Lcd70	L14	MK620121	MK684625	MK684939	Tr_S02	MK619937	MK684778	MK684511			
UR00060	TdF_CC_3	*Lecidea lapicida* (Ach.) Ach.	LP_Lcd70	L14	MK620122	MK684626	MK684940	Tr_S02	MK619938	MK684779	MK684512			
UR00061	TdF_CC_4	*Lecidea auriculata* Th.Fr	LP_Lcd81bb	L18	MK620123	MK684627	MK684941	Tr_S02	MK619939	MK684780	MK684513			
UR00062	TdF_CC_4	*Lecidea auriculata* Th.Fr	LP_Lcd81bb	L18	MK620124	MK684628	MK684942	Tr_S02	MK619940	MK684781	MK684514			
UR00064	TdF_CC_5	*Lecidea lapicida* (Ach.) Ach.	LP_Lcd70	L14	MK620126	MK684629	MK684943	Tr_S02	MK619941	MK684782	MK684515			
UR00065	TdF_CC_5	*Lecidea lapicida* (Ach.) Ach.	LP_Lcd70	L14	MK620126	MK684630		Tr_S02	MK619942	MK684783	MK684516			
UR00067	TdF_CC_6	*Poeltidea perusta* (Nyl.) Hertel & Hafellner	LP_Poe02	POE02	MK620127	MK684631		Tr_S02	MK619943	MK684784	MK684517			
UR00068	TdF_CC_6	*Lecidea lapicida* (Ach.) Ach.	LP_Lcd70	L14	MK620128	MK684632	MK684944	Tr_S02	MK619944	MK684785				
UR00069	TdF_CC_6	*Lecidea lapicida* (Ach.) Ach.	LP_Lcd66	L14	MK620129	MK684633	MK684945	Tr_S02	MK619945	MK684786				
UR00070	TdF_CC_6	*Lecidea medusula* (C.W. Dodge) Hertel	LP_Lcd78	L15	MK620130	MK684634	MK684946	Tr_S02	MK619946	MK684787	MK684518			
UR00073	TdF_VL_1	*Lecidea lapicida* (Ach.) Ach.	LP_Lcd66	L14	MK620131	MK684635	MK684947	Tr_S07	MK619947	MK684788	MK684519			
UR00074	TdF_VL_1	*Poeltidea perusta* (Nyl.) Hertel & Hafellner	LP_Poe02	POE02	MK620132	MK684636		Tr_S02	MK619948	MK684789	MK684520			
UR00075	TdF_VL_2	*Lecidea kalbii* Hertel	LP_Lcd86	L20	MK620133	MK684637	MK684948	Tr_S02	MK619949	MK684790	MK684521			
UR00076	TdF_VL_2	*Lecidea auriculata* Th.Fr.	LP_Lcd81b	L18	MK620134	MK684638		Tr_S02	MK619950	MK684791	MK684522			
UR00077	TdF_VL_2	*Lecidea lapicida* (Ach.) Ach.	LP_Lcd70	L14	MK620135	MK684639		Tr_S02	MK619951	MK684792	MK684523			

**Voucher_ID**	**Location_id**	**Species**	**OTU_id**	**Cluster**	**ITS**	**mtSSU**	**RPB1**	**OTU_id**	**ITS**	**psbJ-L**	**COX2**	**Species**	**OTU_id**	**ITS**
UR00079	TdF_TdF_1	*Porpidia macrocarpa* (DC.) Hertel & A.J.Schwab	LP_Porp20	P08	KX120235	MK684640	MK685055					*A.* sp. URa20	Ast20	KX120196
UR00080	TdF_TdF_2	*Lecidella stigmatea* (Ach.) Hertel & Leuckert	LL_ST15		MK620136			Tr_A04a	MK619952	MK684793				
UR00081	TdF_TdF_2	*Lecidea promiscens* Nyl.	LP_Lcd11	L01	MK620137			Tr_A12	MK619953	MK684794	MK684524			
UR00083	TdF_TdF_3	*Lecidea lapicida* (Ach.) Ach.	LP_Lcd66	L14	MK620138	MK684641		Tr_S02	MK619954	MK684795	MK684525			
UR00084	TdF_TdF_3	*Lecidea lapicida* (Ach.) Ach.	LP_Lcd70	L14	MK620139	MK684642		Tr_S02	MK619955	MK684796	MK684526			
UR00086	RM_MC_1	*Lecidella* sp. 3	LL_EL25		MK620140			Tr_I01j	MK619956					
UR00090	RM_MC_1	*Lecidella stigmatea* (Ach.) Hertel & Leuckert	LL_ST19		MK620141									
UR00091	RM_MC_1	*Lecidea lapicida* (Ach.) Ach.	LP_Lcd66	L14	MK620142	MK684643		Tr_S02	MK619957	MK684797	MK684527			
UR00094	RM_MC_1	*Lecidea lapicida* (Ach.) Ach.	LP_Lcd66	L14	MK620143	MK684644		Tr_S07	MK619958	MK684798	MK684528			
UR00095	RM_MC_1	*Lecidella stigmatea* (Ach.) Hertel & Leuckert	LL_ST08		MK620144			Tr_I17	MK619959	MK684799				
UR00096	RM_MC_1	*Lecidea* sp. UR00096	LP_Lcd65	L13	MK620145			Tr_A37	MK619960	MK684800				
UR00098	RM_MC_1	*Lecidea kalbii* Hertel	LP_Lcd86	L20	MK620146	MK684645	MK684949	Tr_S07	MK619961	MK684801	MK684529			
UR00099	RM_MC_1	*Lecidea cancriformis* (C.W. Dodge & G.E. Baker)	LP_Lcd53	L11	MK620147			Tr_I17	MK619962					
UR00101	RM_MC_1	*Lecidea lapicida* (Ach.) Ach.	LP_Lcd66	L14	MK620148	MK684646	MK684950	Tr_S02	MK619963	MK684802	MK684530			
UR00103	RM_MC_1	*Lecidea lapicida* (Ach.) Ach.	LP_Lcd66	L14	MK620149	MK684647	MK684951	Tr_S07	MK619964					
UR00104	RM_TdP_1	*Lecidea lapicida* (Ach.) Ach.	LP_Lcd66	L14	KX120203	MK684648	MK684952	Tr_S07	MK619965	MK684803	MK684531			
UR00105	RM_TdP_1	*Lecidea lapicida* (Ach.) Ach.	LP_Lcd66	L14	MK620150	MK684649	MK684953	Tr_S07	MK619966	MK684804				
UR00106	RM_TdP_1	*Lecidea lapicida* (Ach.) Ach.	LP_Lcd66	L14	MK620151	MK684650	MK684954	Tr_S07	MK619967	MK684805	MK684532			
UR00107	RM_TdP_1	*Lecidella stigmatea* (Ach.) Hertel & Leuckert	LL_ST20		MK620152			Tr_A38	MK619968	MK684806				
UR00110	RM_TdP_2	*Lecidea cancriformis* (C.W. Dodge & G.E. Baker)	LP_Lcd54	L11	MK620153	MK684651	MK684955	Tr_A39	MK619969	MK684807				
UR00111	RM_TdP_2	*Lecidea lapicida* (Ach.) Ach.	LP_Lcd66	L14	MK620154	MK684652	MK684956	Tr_S16	MK619970	MK684808	MK684533			
UR00114	RM_TdP_2	*Lecidea lapicida* (Ach.) Ach.	LP_Lcd66	L14	MK620155	MK684653	MK684957	Tr_S16	MK619971	MK684809	MK684534			
UR00115	RM_TdP_2	*Lecidea lapicida* (Ach.) Ach.	LP_Lcd66	L14	MK620156	MK684654	MK684958	Tr_S16	MK619972		MK684535			
UR00118	RM_TdP_3	*Lecidea lapicida* (Ach.) Ach.	LP_Lcd66	L14	MK620157	MK684655	MK684959	Tr_S07	MK619973		MK684536			
UR00120	RM_TdP_4	*Lecidella* sp. 3	LL_EL25		MK620158			Tr_A39	MK619974		MK684537			
UR00122	RM_TdP_4	*Lecidea* sp. 4	LP_Lcd64	L12	MK620159	MK684656	MK684960	Tr_A04b	MK619975	MK684810	MK684538			
UR00123	RM_TdP_5	*Porpidia macrocarpa* (DC.) Hertel & A.J.Schwab	LP_Porp24	P08	KX120237	MK684657		Tr_A39	KX120250	MK684811			Ast21	KX120197
UR00124	SC_CC_1	*Lecidea cancriformis* (C.W. Dodge & G.E. Baker)	LP_Lcd55	L11	MK620160	MK684658	MK684961	Tr_I01j	MK619976	MK684812				
UR00126	SC_CC_1	*Lecidea cancriformis* (C.W. Dodge & G.E. Baker)	LP_Lcd55	L11	MK620161	MK684659	MK684962	Tr_A04a	MK619977	MK684813				
UR00127	SC_CC_1	*Lecidea cancriformis* (C.W. Dodge & G.E. Baker)	LP_Lcd55	L11	MK620162	MK684660	MK684963	Tr_A04a	MK619978		MK684539			
UR00128	SC_CC_2	*Lecidella stigmatea* (Ach.) Hertel & Leuckert	LL_ST15		MK620163			Tr_A02	MK619979		MK684540			
UR00129	SC_CC_2	*Lecidea promiscens* Nyl.	LP_Lcd10	L01	KX120205	MK684661	MK684964	Tr_I01j	MK619980					
UR00130	SC_CC_3	*Lecidea* sp. UR00130	LP_Lcd40	L05	MK620164	MK684662	MK684965							
UR00131	SC_CC_3	*Lecidea* sp. 4	LP_Lcd62	L12	MK620165	MK684663	MK684966	Tr_A02	MK619981					

Voucher_ID	Location_id	Species	OTU_id	Cluster	ITS	mtSSU	RPB1	OTU_id	ITS	psbJ-L	COX2	Species	OTU_id	ITS
UR00133	SC_CC_4	*Lecidella stigmatea* (Ach.) Hertel & Leuckert	LL_ST07		MK620166									
UR00134	SC_CC_4	*Lecidea cancriformis* (C.W. Dodge & G.E. Baker)	LP_Lcd57	L11	MK620167	MK684664	MK684967	Tr_I01i	MK619982					
UR00135	SC_CC_5	*Lecidea* sp. 3	LP_Lcd29	L04	KX120209	MK684665	MK684968	Tr_A12	MK619983	MK684814				
UR00137	SC_LP_1	*Lecidea* sp. 3	LP_Lcd29	L04	MK620168	MK684666	MK684969	Tr_A12	MK619984	MK684815				
UR00138	SC_LP_1	*Lecidea tesselata* Flörke	LP_Lcd85	L18	KX120213	MK684667	MK684970	Tr_I17	MK619985	MK684816				
UR00139	SC_LP_1	*Lecidea tesselata* Flörke	LP_Lcd85	L18	MK620169	MK684668	MK684971	Tr_I17	MK619986	MK684817				
UR00141	SC_LP_2	*Lecidea* sp. UR00141	LP_Lcd84	L18	MK620170	MK684669	MK684972	Tr_I17	MK619987					
UR00142	SC_LP_3	*Lecidea lapicida* (Ach.) Ach.	LP_Lcd66	L14	MK620171	MK684670	MK684973	Tr_S07	MK619988	MK684818	MK684541			
UR00143	SC_LP_3	*Lecidea* sp. 4	LP_Lcd63	L12	MK620172	MK684671	MK684974	Tr_I17	MK619989	MK684819				
UR00144	SC_LP_3	*Lecidea* sp. 4	LP_Lcd63	L12	MK620173	MK684672	MK684975	Tr_I17	MK619990	MK684820				
UR00145	SC_LP_3	*Lecidea* sp. 4	LP_Lcd62	L12	MK620174	MK684673	MK684976	Tr_I17	MK619991	MK684821				
UR00146	SC_LP_3	*Lecidea lapicida* (Ach.) Ach.	LP_Lcd66	L14	MK620175	MK684674		Tr_S07	MK619992		MK684542			
UR00147	SC_LP_4	*Lecidea cancriformis* (C.W. Dodge & G.E. Baker)	LP_Lcd57	L11	MK620176	MK684675		Tr_I17	MK619993	MK684822				
UR00148	SC_LP_4	*Lecidea cancriformis* (C.W. Dodge & G.E. Baker)	LP_Lcd52	L11	MK620177			Tr_S02	MK619994					
UR00149	SC_LP_4	*Lecidea promiscens* Nyl.	LP_Lcd12	L01	MK620178	MK684676	MK684977	Tr_I01j	MK619995	MK684823				
UR00150	SC_LP_4	*Lecidea promiscens* Nyl.	LP_Lcd10	L01	MK620179	MK684677	MK684978	Tr_I01j	MK619996	MK684824				
UR00151	SC_LP_4	*Lecidea promiscens* Nyl.	LP_Lcd10	L01	MK620180	MK684678	MK684979	Tr_S02	MK619997	MK684825				
UR00152	SC_LP_5	*Lecidea promiscens* Nyl.	LP_Lcd10	L01	MK620181	MK684679	MK684980	Tr_A36	MK619998	MK684826	MK684543			
UR00153	SC_LP_6	*Lecidea cancriformis* (C.W. Dodge & G.E. Baker)	LP_Lcd57	L11	MK620182	MK684680	MK684981	Tr_I17	MK619999					
UR00154	SC_LP_6	*Lecidea cancriformis* (C.W. Dodge & G.E. Baker)	LP_Lcd57	L11	MK620183	MK684681	MK684982	Tr_S02	MK620000	MK684827				
UR00155	SC_LP_6	*Lecidea cancriformis* (C.W. Dodge & G.E. Baker)	LP_Lcd57	L11	MK620184	MK684682		Tr_S02	MK620001	MK684828				
UR00156	SC_LP_6	*Lecidea cancriformis* (C.W. Dodge & G.E. Baker)	LP_Lcd56	L11	MK620185	MK684683	MK684983	Tr_I17	MK620002					
UR00157	SC_LP_6	*Lecidella stigmatea* (Ach.) Hertel & Leuckert	LL_ST09		MK620186			Tr_A02	MK620003					
UR00158	SC_LP_6	*Lecidea* sp. 2 (T43427)	LP_Lcd18	L03	MK620187	MK684684	MK684984	Tr_S02	MK620004	MK684829				
UR00162	SC_LT_1	*Lecidella stigmatea* (Ach.) Hertel & Leuckert	LL_ST17		MK620188			Tr_A38	MK620005	MK684830	MK684544			
UR00163	SC_LT_2	*Lecidella stigmatea* (Ach.) Hertel & Leuckert	LL_ST16		MK620189			Tr_A38	MK620006	MK684831	MK684545			
UR00164	SC_LT_2	*Lecidella stigmatea* (Ach.) Hertel & Leuckert	LL_ST18		MK620190			Tr_A38	MK620007	MK684832	MK684546			
UR00166	SC_LT_3	*Lecidea cancriformis* (C.W. Dodge & G.E. Baker)	LP_Lcd54	L11	MK620191	MK684685		Tr_A04b	MK620008	MK684833	MK684547			
UR00170	SC_LT_4	*Lecidea auriculata* Th.Fr	LP_Lcd81b	L18	MK620192	MK684686	MK684985	Tr_S02	MK620009	MK684834				
UR00171	SC_LT_5	*Lecidea lapicida* (Ach.) Ach.	LP_Lcd66	L14	MK620193	MK684687	MK684986	Tr_S02	MK620010	MK684835				
UR00173	SC_LT_5	*Lecidea auriculata* Th.Fr	LP_Lcd81b	L18	MK620194	MK684688	MK684987	Tr_S07	MK620011	MK684836				
UR00174	SC_LT_6	*Lecidea* sp. 2 (T43427)	LP_Lcd18	L03	MK620195	MK684689	MK684988	Tr_S02	MK620012	MK684837				
UR00175	SC_LT_7	*Lecidea auriculata* Th.Fr.	LP_Lcd81b	L18	MK620196	MK684690	MK684989	Tr_S02	MK620013	MK684838				
UR00176	SC_LT_7	*Lecidea lapicida* (Ach.) Ach.	LP_Lcd66	L14	MK620197	MK684691		Tr_S02	MK620014	MK684839				

Voucher_ID	Location_id	Species	OTU_id	Cluster	ITS	mtSSU	RPB1	OTU_id	ITS	psbJ-L	COX2	Species	OTU_id	ITS
UR00177	SC_LT_7	*Lecidea* sp. 2 (T43427)	LP_Lcd18	L03	KX120211	MK684692	MK684990	Tr_S02	MK620015	MK684840	MK684548			
UR00179	CH_TC_1	*Lecidea cancriformis* (C.W. Dodge & G.E. Baker)	LP_Lcd52	L11	MK620198	MK684693	MK684991	Tr_A39	MK620016	MK684841				
UR00181	CH_TC_1	*Lecidea kalbii* Hertel	LP_Lcd87	L20	MK620199	MK684694	MK684992	Tr_S07	MK620017	MK684842	MK684549			
UR00183	CH_TC_1	*Lecidea cancriformis* (C.W. Dodge & G.E. Baker)	LP_Lcd54	L11	MK620200	MK684695	MK684993	Tr_A39	MK620018	MK684843				
UR00184	CH_TC_1	*Lecidea kalbii* Hertel	LP_Lcd87	L20	MK620201	MK684696	MK684994	Tr_S02	MK620019	MK684844				
UR00186	CH_TC_1	*Lecidea lapicida* (Ach.) Ach.	LP_Lcd66	L14	MK620202	MK684697	MK684995	Tr_S02	MK620020	MK684845				
UR00188	CH_TC_1	*Lecidea tesselata* Flörke	LP_Lcd85	L18	MK620203	MK684698	MK684996	Tr_A04b	MK620021	MK684846	MK684550			
UR00189	CH_TC_1	*Lecidea cancriformis* (C.W. Dodge & G.E. Baker)	LP_Lcd54	L11	MK620204	MK684699	MK684997	Tr_A04b	MK620022	MK684847				
UR00190	CH_TC_1	*Lecidea cancriformis* (C.W. Dodge & G.E. Baker)	LP_Lcd52	L11	MK620205	MK684700	MK684998	Tr_A04b	MK620023	MK684848	MK684551			
UR00192	CH_TC_1	*Lecidea lapicida* (Ach.) Ach.	LP_Lcd66	L14	MK620206	MK684701	MK684999	Tr_S02	MK620024	MK684849				
UR00193	CH_AL_1	*Porpidia macrocarpa* (DC.) Hertel & A.J.Schwab	LP_Porp25	P08	KX120236	MK684702		Tr_S07	MK620025	MK684850		*A. woessiae*	Ast13	KX120199
UR00194	CH_LH_1	*Lecidea* sp. 2 (T43427)	LP_Lcd18	L03	MK620207	MK684703	MK685000	Tr_S02	MK620026	MK684851				
UR00195	CH_LH_1	*Lecidea* sp. 3	LP_Lcd27	L04	KX120210	MK684704	MK685001	Tr_A12	MK620027	MK684852	MK684552			
UR00196	CH_LH_1	*Lecidea* sp. 1	LP_Lcd09a	L01	KX120208	MK684705	MK685002	Tr_I01j	MK620028	MK684853				
UR00198	CH_LH_2	*Lecidea lapicida* (Ach.) Ach.	LP_Lcd66	L14	MK620208	MK684706	MK685003	Tr_S02	MK620029	MK684854				
UR00199	CH_LH_2	*Lecidea medusula* (C.W. Dodge) Hertel	LP_Lcd78	L15	MK620209	MK684707	MK685004	Tr_S02	MK620030	MK684855				
UR00200	CH_LH_3	*Lecidea atrobrunnea* (Ramond ex Lam. DC.) Schaer.	LP_Lcd01	L01	KX120206	MK684708	MK685005	Tr_I01j	MK620031					
UR00201	CH_LH_3	*Lecidea promiscens* Nyl.	LP_Lcd10	L01	MK620210	MK684709	MK685006	Tr_I01j	MK620032					
UR00202	CH_LH_3	*Lecidea* sp. 1	LP_Lcd09a	L01	MK620211	MK684710	MK685007	Tr_S02	MK620033	MK684856				
UR00204	CH_LH_3	*Lecidea promiscens* Nyl.	LP_Lcd10	L01	MK620212	MK684711	MK685008	Tr_I01j	MK620034	MK684857				
UR00205	CH_LH_4	*Lecidea lapicida* (Ach.) Ach.	LP_Lcd66	L14	MK620213	MK684712	MK685009	Tr_S02	MK620035	MK684858				
UR00206	CH_LH_4	*Lecidea auriculata* Th.Fr.	LP_Lcd81b	L18	MK620214	MK684713	MK685010	Tr_S02	MK620036	MK684859	MK684553			
UR00207	CH_LH_4	*Lecidea auriculata* Th.Fr.	LP_Lcd81b	L18	MK620215	MK684714	MK685011	Tr_S07	MK620037	MK684860				
UR00208	CH_LH_4	*Lecidella stigmatea* (Ach.) Hertel & Leuckert	LL_ST09		MK620216			Tr_A02	MK620038					
UR00209	CH_LH_4	*Lecidea auriculata* Th.Fr.	LP_Lcd81b	L18	MK620217	MK684715	MK685012	Tr_S02	MK620039	MK684861				
UR00210	CH_LH_5	*Lecidella* sp. 1	LL_N01		MK620218			Tr_I01j	MK620040	MK684862				
UR00211	CH_LH_5	*Lecidella* sp. 1	LL_N01		MK620219			Tr_I01j	MK620041	MK684863				
UR00212	CH_LH_5	*Lecidea atrobrunnea* (Ramond ex Lam. DC.) Schaer.	LP_Lcd05	L01	KX120207	MK684716	MK685013	Tr_I01j	MK620042	MK684864				
UR00213	CH_LH_5	*Lecidella* sp. 1	LL_N01		MK620221			Tr_S02	MK620043	MK684865	MK684554			
UR00214	CH_LH_5	*Lecidea atrobrunnea* (Ramond ex Lam. DC.) Schaer.	LP_Lcd01	L01	MK620222	MK684717	MK685014	Tr_I01j	MK620044	MK684866				
UR00215	CH_LH_5	*Lecidea promiscens* Nyl.	LP_Lcd10	L01	MK620223	MK684718	MK685015	Tr_I01j	MK620045					
UR00216	CH_LH_5	*Lecidea* sp. 1	LP_Lcd09a	L01	MK620224	MK684719	MK685016	Tr_I01j	MK620046	MK684867				
UR00217	CH_LH_5	*Lecidea* sp. 1	LP_Lcd09a	L01	MK620225	MK684720	MK685017	Tr_I01j	MK620047	MK684868				
UR00218	CH_LH_5	*Lecidea* sp. 3	LP_Lcd27	L04	MK620226	MK684721	MK685018	Tr_I01j	MK620048	MK684869				

Voucher_ID	Location_id	Species	OTU_id	Cluster	ITS	mtSSU	RPB1	OTU_id	ITS	psbJ-L	COX2	Species	OTU_id	ITS
UR00219	CH_LH_5	*Lecidea* sp. 1	LP_Lcd09a	L01	MK620227	MK684722	MK685019	Tr_I01j	MK620049	MK684870				
UR00220	CH_CC_1	*Lecidea cancriformis* (C.W. Dodge & G.E. Baker)	LP_Lcd54	L11	MK620228	MK684723		Tr_A04b	MK620050	MK684871	MK684555			
UR00221	RN_CC_1	*Lecidea* sp. 2 (T43427)	LP_Lcd18	L03	MK620229	MK684724	MK685020	Tr_S07	MK620051	MK684872				
UR00222	RN_CC_1	*Lecidea* sp. 1	LP_Lcd09a	L01	MK620230	MK684725	MK685021	Tr_S02	MK620052	MK684873				
UR00224	RN_CC_1	*Lecidea auriculata* Th.Fr.	LP_Lcd81b	L18	MK620231	MK684726		Tr_S07	MK620053	MK684874	MK684556			
UR00227	RN_CC_2	*Lecidea* sp. 2 (T43427)	LP_Lcd18	L03	MK620232	MK684727	MK685022	Tr_S02	MK620054	MK684875	MK684557			
UR00228	RN_CC_2	*Lecidea lapicida* (Ach.) Ach.	LP_Lcd70	L14	MK620233	MK684728	MK685023	Tr_S02	MK620055	MK684876	MK684558			
UR00229	RN_CC_2	*Lecidea lapicida* (Ach.) Ach.	LP_Lcd70	L14	MK620234	MK684729	MK685024	Tr_S02	MK620056	MK684877	MK684559			
UR00230	RN_CC_2	*Lecidea auriculata* Th.Fr.	LP_Lcd81b	L18	MK620235	MK684731	MK685025	Tr_S02	MK620057		MK684560			
UR00231	RN_CC_3	*Lecidea lapicida* (Ach.) Ach.	LP_Lcd66	L14	MK620236	MK684732	MK685026	Tr_S02	MK620058	MK684878	MK684561			
UR00232	RN_CC_3	*Lecidea lapicida* (Ach.) Ach.	LP_Lcd76	L14	MK620237	MK684733	MK685027	Tr_S07	MK620059		MK684562			
UR00233	RN_CC_3	*Lecidea* sp. 1	LP_Lcd09b	L01	MK620238	MK684734	MK685028	Tr_S02	MK620060	MK684879	MK684563			
UR00234	RN_CC_3	*Lecidea lapicida* (Ach.) Ach.	LP_Lcd66	L14	MK620239	MK684735	MK685029	Tr_S02	MK620061	MK684880	MK684564			
UR00235	RN_CC_3	*Lecidea atrobrunnea* (Ramond ex Lam. DC.) Schaer.	LP_Lcd01	L01	MK620240	MK684736	MK685030	Tr_I01i	MK620062	MK684881				
UR00236	RN_CC_3	*Lecidea* sp. 1	LP_Lcd09b	L01	MK620241	MK684737	MK685031	Tr_A37	MK620063					
UR00237	RN_CC_3	*Lecidea lapicida* (Ach.) Ach.	LP_Lcd66	L14	MK620242	MK684738	MK685032	Tr_S02	MK620064	MK684882	MK684565			
UR00242	RN_CC_4	*Lecidea* sp. 2 (T43427)	LP_Lcd18	L03	MK620243	MK684739	MK685033	Tr_S02	MK620065	MK684883	MK684566			
UR00243	RN_CC_4	*Lecidea* sp. 2 (T43427)	LP_Lcd18	L03	MK620244	MK684740	MK685034	Tr_S02	MK620066	MK684884	MK684567			
UR00244	RN_CC_4	*Lecidea promiscens* Nyl.	LP_Lcd10	L01	MK620245	MK684741	MK685035	Tr_I01i	MK620067					
UR00245	RN_CC_4	*Lecidea auriculata* Th.Fr.	LP_Lcd81b	L18	MK620246	MK684742	MK685036	Tr_S02	MK620068	MK684885	MK684568			

S1-2b Additional photobiont (*Asterochloris/Vulcanochloris, Trebouxia*) sequences including newly generated accessions (marked in bold) and those downloaded from GenBank (NCBI) worldwide. The representative sequences for the *Trebouxia* OTUs, as defined by Leavitt *et al.* (2015) that were additionally included in the phylogeny (*Trebouxia* - ITS, Figure 6) are not mentioned in the table. Taxonomic information was taken from the following literature: Leavitt *et al.* (2015); Moya *et al.* (2015); Skaloud *et al.* (2015); Vancurova *et al.* (2015).

Voucher_id	Country	Algal species	OTU_id	Accession number ITS
	Italy	*Asterochloris phycobionta* Tschermak-Woess	Ast01	AM900490
	Italy, Trento	*A. phycobionta* Tschermak-Woess	Ast01	GU017647
	Czech Rep., Vladar Mt.	*A. lobophora* Skaloud & Peksa	Ast01	AM906006
	Slovakia, Povazsky Inovec Mts.	*A. lobophora* Skaloud & Peksa	Ast01	KP318672
	USA, Florida	*Asterochloris* sp. RY666	Ast01	AY712702
	USA, Arkansas	*Asterochloris* sp. RY1160	Ast01	DQ482674
	Czech Rep., Sumava Mts.	*A. leprarii* Skaloud & Peksa	Ast02	AM906002

Voucher_id	Country	Algal species	OTU_id	Accession number ITS
T51483 (SZU)	Austria, Salzburg	*Asterochloris* sp. URa14	Ast03	KF907660
T51478 (SZU)	Austria, Salzburg	*Asterochloris* sp. URa14	Ast03	KF907659
T51474 (SZU)	Austria, Salzburg	*Asterochloris* sp. URa14	Ast03	KF907657
	USA, Vermont	*A. excentrica* UTEX1714 (Archibald) Skaloud & Peksa	Ast04	AM905993
	Czech Rep., Luzicke hory Mts.	*A. echinata* Skaloud & Peksa	Ast06	FM955666
	USA	*Asterochloris* sp. MN107	Ast06	AF345434
	Spain, Alicante	*A. mediterranea* E.Barreno, S.Chiva, P.Moya & P.Škaloud	Ast06	KP257384
	Sweden, Öland	*Asterochloris* sp. DrostorpA1	Ast06	KT215300
	Sweden, Öland	*Asterochloris* sp. GyngeD8b	Ast06	KT215312
	Yugoslavia	*Asterochloris* sp. I03	Ast06	AF345435
	Spain, Lancerote	*A. mediterranea* E.Barreno, S.Chiva, P.Moya & P.Škaloud	Ast06	KP257394
	Spain, Tarragona	*Asterochloris* sp. MACB 90574	Ast06	FM205725
	Sweden, Oland	*Asterochloris* sp. GyngeA9	Ast06	KT215309
		Trebouxia sp. s45553	Ast07	FJ756789
	Australia	*Asterochloris* sp. 7090	Ast08	AF345424
	India, Himachal Pradesch		Ast09	HE803030
	Czech Rep., Snezik Mts.	*A. gaertneri* Skaloud & Peksa	Ast10	AM905997
	Italy	*Asterochloris* sp. MN03	Ast11	AF345436
		Asterochloris sp K1028	Ast12	FJ756801
	India: Uttarakhand		Ast12	HE803050
		Asterochloris sp. K1091	Ast12	FJ756793
	Spain, Somosierra	*A. woessiae* Skaloud & Peksa	Ast13	KP318669
	Slovakia, Gelnica	*A. irregularis* (Hildreth & Ahmadijan) Skaloud & Peksa	Ast14	AM906000
US12432c (SZU)	Norway, Svalbard	*A. irregularis* (Hildreth & Ahmadijan) Skaloud & Peksa	Ast14	MK620072
	Czech Rep., Studenec Mts.	*A. glomerata* (Warén) Skaloud & Peksa	Ast14	AM905998
	USA, Whitinsville, MA	*A. erici* UTEX 910 (Ahmadijan) Skaloud & Peksa	Ast15	AF345439
	Canada		Ast16	FJ553359
T51469 (SZU)	Sweden, Öland	*Asterochloris* sp. URa16	Ast17	KF907654
T51494 (SZU)	Germany, Bayern	*Asterochloris* sp. URa16	Ast17	KF907663
T51493 (SZU)	Germany, Bayern	*Asterochloris* sp. URa16	Ast17	KF907662
T51495 (SZU)	Germany, Bayern	*Asterochloris* sp. URa16	Ast17	KF907664
T51477 (SZU)	Austria, Salzburg	*Asterochloris* sp. URa15	Ast18	KF907658
T51487 (SZU)	Austria, Salzburg	*Asterochloris* sp. URa15	Ast18	KF907661
	USA, Washington	*A. magna* (Archibald) Skaloud & Peksa	Ast19	AM906012
T51501 (SZU)	Germany, Bayern	*A. magna* (Archibald) Skaloud & Peksa	Ast19	KF907669
Ps01/GÖSS-001*	Germany, Bayern	*Vulcanochloris* sp. URa17	Vul01	KF907645
T51496 (SZU)	Germany, Bayern	*Vulcanochloris* sp. URa17	Vul01	KF907665
	Spain, La Plama	*V. guanchorum* Vancurová, Peksa, Nemcová & Skaloud	Vul01	KR952330
	Spain, La Plama	*V. symbiotica* Vancurová, Peksa, Nemcová & Skaloud	Vul02	KR952331
	Spain, La Plama	*V. canariensis* Vancurová, Peksa, Nemcová & Skaloud	Vul03	KR952320
	Spain, La Plama	*V. canariensis* Vancurová, Peksa, Nemcová & Skaloud	Vul04	KR952329

Voucher_id	Country	Algal species	OTU_id	Accession number ITS
T35895 (SZU)	Antarctica, S. Victoria Land	*Trebouxia* sp. URa2	Tr_A02	JN204757
T44641 (SZU)	Antarctica, Darwin Area	*Trebouxia* sp. URa3	Tr_A04	JN204803
T51458 (SZU)	Spain, Almeria	*Trebouxia* sp. URa8	Tr_A15	KF907531
Ps06/ALM-071*	Spain, Almeria	*Trebouxia* sp. URa16	Tr_A35	KF907523
T51465 (SZU)	Spain, Almeria	*Trebouxia* sp. URa16	Tr_A35	KF907525
T51457 (SZU)	Spain, Almeria	*Trebouxia* sp. URa10	Tr_A40	KF907529
T33712 (SZU)	Antarctica, S. Victoria Land	*Trebouxia* sp. URa2	Tr_A41	JN204744
US12426b (SZU)	Norway, Svalbard		Tr_I01i	MK620071
T43008 (SZU)	Antarctica, S. Victoria Land		Tr_I01j	JN204778
US12399 (SZU)	Norway, Svalbard		Tr_I16	MK620070
T35647 (SZU)	Antarctica, Darwin Area		Tr_S02	JN204751
Ps019/GYN-051*	Sweden, Öland		Tr_S02	KF907553
T44655 (SZU)	Antarctica, Darwin Area		Tr_S02b	MK226915
T44693 (SZU)	Antarctica, Darwin Area		Tr_S02b	JN204810
T44717 (SZU)	Antarctica, Darwin Area		Tr_S02b	MK226949
T44720 (SZU)	Antarctica, Darwin Area		Tr_S02c	MK226951
	United Kingdom, Scotland		Tr_S03	KT827678
	Ukraine		Tr_S08	KT827698
	Bolivia		Tr_S10	KT827666
Ps012/Hoch-042*	Austria, Salzburg	*Trebouxia* sp. URa4	Tr_S12	KF907551
	Turkey		Tr_S14	KT827706
Ps013/Hoch-038*	Austria, Salzburg	*Trebouxia* sp. URa11	Tr_S14	KF907534
US12398 (SZU)	Norway, Svalbard		Tr_S15	MK620069
	Kenya		Tr_S17	KT827704
T42990 (SZU)	Antarctica, Darwin Area	*Trebouxia* sp. URa1	Tr_S18	JN204770
Ps020/GYN-006*	Sweden, Öland	*Trebouxia* sp. URa12	Tr_S19	KF907554
	Iceland		Tr_S20	MG098085
	Iceland		Tr_S21	MG098082
	Iceland		Tr_S22	MG098091
	Iceland		Tr_S23	MG098086

S1-2c Additional mycobiont (*Lecidella, Lecidea, Porpidia, Cyclohymenia*) sequences including newly generated (marked in bold) accessions and those downloaded from GenBank worldwide. Taxonomic information was taken from the following literature: Ruprecht *et al.* (2010, 2012*b*); Wirth *et al.* (2013); Orange (2014); Zhao *et al.* (2015); Hafellner & Türk (2016); McCune *et al.* (2017).

Voucher_id	Country	Mycobiont species	OTU_id	Cluster	Accession number		
					ITS	mtSSU	RBP1
	China, Xinjiang	*Lecidella* aff. *elaeochroma* (Ach.) Choisy	LL_EL01		KT453751		
	China, Xinjiang	*L.* aff. *elaeochroma (Ach.) Choisy*	LL_EL02		KT453752		
	Finland	*L. euphorea* (Flörke) Hertel	LL_EL03		HQ650596		
	China, Xinjiang	*L. tumidula* (A. Massal.) Knoph & Leuckert	LL_EL13		KT453736		
	China, Xinjiang	*L. tumidula* (A. Massal.) Knoph & Leuckert	LL_EL13		KT453737		
	China, Inner Mongolia	*L. carpathica* Körb.	LL_EL04		KT453739		
	China, Xinjiang	*L. carpathica* Körb.	LL_EL06		KT453741		
	China, Xinjiang	*L. carpathica* Körb.	LL_EL13		KT453740		
	China, Yunnan	*L. effugiens* (Nilson) Knoph & Hertel	LL_EL08		KT453754		
	China, Yunnan	*L.* aff. *euphorea* (Flörke) Hertel	LL_EL09		KT453755		
	China, Shaanxi	*L.* aff. *euphorea* (Flörke) Hertel	LL_EL10		KT453756		
	China, Xinjiang	*L. elaeochromoides* (Nyl.) Knoph & Hertel	LL_EL08		KT453746		
	China, Xinjiang	*L. effugiens* (Nilson) Knoph & Hertel	LL_EL08		KT453748		
	China, Xinjiang	*L. elaeochromoides* (Nyl.) Knoph & Hertel	LL_EL12		KT453750		
	China, Xinjiang	*L. elaeochroma* (Ach.) Choisy	LL_EL13		KT453749		
	Sweden	*L. meiococca* (Nyl.) Leuckert & Hertel	LL_EL12		AF517929		
		L. elaeochroma (Ach.) Choisy	LL_EL13		AY541275		
	Hungary	*L. elaeochroma* (Ach.) Choisy	LL_EL16		EU266082		
	Turkey, Trabazon	*L. elaeochroma* (Ach.) Choisy	LL_EL16		HQ605936		
	Turkey, Trabazon	*L. elaeochroma* (Ach.) Choisy	LL_EL13		HQ605938		
	United Kingdom	*L. elaeochroma* (Ach.) Choisy	LL_EL13		FR799217		
	China, Xinjiang	*L. euphorea* (Flörke) Hertel	LL_EL18		KT453742		
	China, Xinjiang	*L. euphorea* (Flörke) Hertel	LL_EL18		KT453743		
	China, Xinjiang	*L. euphorea* (Flörke) Hertel	LL_EL18		KT453744		
	China, Xinjiang	*L. euphorea* (Flörke) Hertel	LL_EL13		KT453745		
T39666 (SZU)	Austria, Salzburg	*L. wulfenii* (Hepp) Körb	LL_EL13		JN873903		
	United Kingdom	*L. flavosorediata* (Vězda) Hertel & Leuckert	LL_EL21		FR799213		
	United Kingdom	*L. elaeochroma* (Ach.) Choisy	LL_EL22		FR799214		
	Belgium	*L. elaeochroma* (Ach.) Choisy	LL_EL22		HQ650605		
	United Kingdom	*L. elaeochroma* (Ach.) Choisy	LL_EL18		FR799215		
	United Kingdom	*L. elaeochroma* (Ach.) Choisy	LL_EL18		FR799216		

Voucher_id	Country	Mycobiont species	OTU_id	Cluster	ITS	mtSSU	RBP1
	China, Shaanxi	*L. enteroleucella* (Nyl.) Hertel	LL_EN01		KT453757		
**MAF_GS1_64**	Antarctica, Transantarctic Mts.	*Lecidella* sp. 2	LL_N02		MK208746		
T38608 (SZU)	Austria	*L. stigmatea* (Ach.) Hertel & Leuckert	LL_ST01		JN873901		
	Turkey	*L. stigmatea* (Ach.) Hertel & Leuckert	LL_ST02		HQ605937		
	China	*L. stigmatea* (Ach.) Hertel & Leuckert	LL_ST02		KT453766		
U.Ruprecht48 (SZU)	Austria	*L. patavina* (A.Massal.) Knoph & Leuckert	LL_ST02		JN873894		
T39655 (SZU)	Austria	*L. patavina* (A.Massal.) Knoph & Leuckert	LL_ST03		JN873893		
T39665 (SZU)	Austria	*L. stigmatea* (Ach.) Hertel & Leuckert	LL_ST03		JN873902		
	China	*L. patavina* (A.Massal.) Knoph & Leuckert	LL_ST04		KT453767		
	Turkey	*L. patavina* (A.Massal.) Knoph & Leuckert	LL_ST05		HQ605934		
	China	*L. stigmatea* (Ach.) Hertel & Leuckert	LL_ST06		KT453768		
T43015 (SZU)	Antarctica, S. Victoria Land	*L. greenii* U. Ruprecht & Türk	LL_ST10		HQ287871		
T33586 (SZU)	Antarctica, S. Victoria Land	*L. greenii* U. Ruprecht & Türk	LL_ST11		JN873883		
T32991 (SZU)	Antarctica, S. Victoria Land	*L. siplei* (C.W. Dodge & G.E. Baker) May. Inoue	LL_ST12		JN873896		
T33457 (SZU)	Antarctica, S. Victoria Land	*L. siplei* (C.W. Dodge & G.E. Baker) May. Inoue	LL_ST13		JN873898		
	China	*L. patavina* (A.Massal.) Knoph & Leuckert	LL_ST14		KT453761		
	China, Xinjiang	*L. stigmatea* (Ach.) Hertel & Leuckert	LL_ST16		KT453759		
	China	*L. stigmatea* (Ach.) Hertel & Leuckert	LL_ST18		KT453758		
	China	*L. stigmatea* (Ach.) Hertel & Leuckert	LL_ST18		KT453760		
	China	*L. stigmatea* (Ach.) Hertel & Leuckert	LL_ST18		KT453762		
	China	*L. stigmatea* (Ach.) Hertel & Leuckert	LL_ST18		KT453763		
**AAS_Convey00458**	Antarctic Peninsula	*Lecidea atrobrunnea* (Ramond ex Lam. DC.) Schaer.	LP_Lcd01	L01	MK620076	MK684569	MK684891
**AAS_Smith12030**	Antarctic Peninsula	*L. atrobrunnea* (Ramond ex Lam. DC.) Schaer.	LP_Lcd01	L01	MK620079	MK684571	MK684893
LE_A060124	Antarctic Peninsula	*L. atrobrunnea* (Ramond ex Lam. DC.) Schaer.	LP_Lcd01	L01	GU074457	GU074505	MK684899
Leavitt 17.518 (BRY-C)	USA, Nevada	*L. atrobrunnea* (Ramond ex Lam. DC.) Schaer. [*L. syncarpa* Zahlbr.]	LP_Lcd01	L01	MK990107		
	Turkey, Trabazon	*L. atrobrunnea* (Ramond ex Lam. DC.) Schaer. [*L. syncarpa* Zahlbr.]	LP_Lcd02	L01	KF570277		
UCR174803	USA, California	*L. atrobrunnea* ssp. *saxosa* Hertel & Leuckert (Ramond) Schaer.	LP_Lcd03	L01	GU074456	GU074507	MK684918
Lund_LG770	Greenland	*L. atrobrunnea* (Ramond ex Lam. DC.) Schaer.	LP_Lcd04	L01	EU259897	GU074510	MK684906
**LE_A060137**	Antarctic Peninsula	*L. atrobrunnea* (Ramond ex Lam. DC.) Schaer.	LP_Lcd05	L01	MK620083	MK684573	MK684900
**US12406 (SZU)**	Norway, Svalbard	*L. atrobrunnea* (Ramond ex Lam. DC.) Schaer.	LP_Lcd06	L01	MK620265		
Leavitt 16.604 (BRY-C)	USA, Utah	*L. atrobrunnea* (Ramond ex Lam. DC.) Schaer.	LP_Lcd07	L01	MK990105		
T34435 (SZU)	Austria, Kärnten	*L. atrobrunnea* ssp. *stictica* Hertel & Leuckert (Ramond) Schaer.	LP_Lcd08	L01	EU259899	GU074506	MK684908
**UR00471 (SZU)**	Austria, Salzburg	*L. promiscens* Nyl.	LP_Lcd13	L01	MK620263		
**MAF_LI_2a (SZU)**	Antarctica, Livingston Island	*L. promiscens* Nyl.	LP_Lcd14	L01	MK620091		
**UR00422 (SZU)**	Austria, Salzburg	*L. swartzioidea* Nyl.	LP_Lcd15	L01	MK620260		
T39641 (SZU)	Austria	*L. confluens* (Weber) Ach.	LP_Lcd16	L02	EU263921	GU074492	
	Switzerland	*Lecidea* sp. (*P. speirea*)	LP_Lcd17	L02	HQ650631	DQ986865	DQ986829.1
**LE_A111504**	Antarctica, Enderby Land	*Lecidea* sp. 2	LP_Lcd18	L03	MK620085	MK684575	MK684902
T43427 (SZU)	Norway, Svalbard	*Lecidea* sp. 2	LP_Lcd18	L03	GU074426	GU074502	MK684917
Leavitt 18.148 (BRY-C)	USA, Utah	*L.* cf. *polypycnidophora* U. Rupr. & Türk	LP_Lcd19	L04	MK990117		
Leavitt 18-856 (BRY-C)	USA, Utah	*L.* cf. *polypycnidophora* U. Rupr. & Türk	LP_Lcd20	L04	MK990110		

Voucher_id	Country	Mycobiont species	OTU_id	Cluster	ITS	mtSSU	RBP1
LOSD #1165	USA, South Dakota	*L.* cf. *polypycnidophora* U. Rupr. & Türk	LP_Lcd21	L04	EU263929	GU074501	MK684907
	China	*L.* cf. *polypycnidophora* U. Rupr. & Türk	LP_Lcd21	L04	MH231446		
**LE_A0511701**	Antarctica, MacRobertson Land	*L. polypycnidophora* U. Rupr. & Türk	LP_Lcd22	L04	MK620081	MK684572	MK684897
Leavitt 18-597 (BRY-C)	USA, Utah	*L.* '*andersonii* agg. sp 2'	LP_Lcd23	L04	MK990111		
Leavitt 17.525 (BRY-C)	USA, Nevada	*L.* '*andersonii* agg. sp 1'	LP_Lcd24	L04	MK990109		
LE_A0515601	Antarctica, Dronning Maud Land	*L. andersonii* Filson	LP_Lcd25	L04	GU074445	GU074469	MK684898
T43425 (SZU)	Norway, Svalbard	*L. andersonii* Filson	LP_Lcd25	L04	GU074442	GU074467	MK684915
T43003	Antarctica, S. Victoria Land	*L. andersonii* Filson	LP_Lcd25	L04	GU074451	GU074461	MK684912
LE_A105701	Antarctica, Dronning Maud Land	*L. andersonii* Filson	LP_Lcd25	L04	MK620084	MK684574	MK684901
Leavitt 17.672 (BRY-C)	USA, Nevada	*L. andersonii* Filson	LP_Lcd25	L04	MK990112		
**LE_A133003**	Antarctica, MacRobertson Land	*Lecidea* sp. UCR1	LP_Lcd26	L04	MK620086	GU074475	MK684903
Leavitt 17-529 (BRY-C)	USA, Nevada	*Lecidea* sp. 3	LP_Lcd27	L04	MK990123		
Leavitt 18-637 (BRY-C)	USA, Utah	*Lecidea* sp. 3	LP_Lcd28	L04	MK990126		
Leavitt 18-297 (BRY-C)	USA, Utah	*Lecidea* sp. 3	LP_Lcd29	L04	MK990121		
Leavitt 18-602 (BRY-C)	USA, Utah	*Lecidea* sp. 3	LP_Lcd30	L04	MK990118		
Leavitt 17.524 (BRY-C)	USA, Utah	*Lecidea* sp. 3	LP_Lcd31	L04	MK990115		
Leavitt 18-292 (BRY-C)	USA, Utah	*Lecidea* sp. 3	LP_Lcd32	L04	MK990116		
	USA, California	*L. violascens* H. Magn.	LP_Lcd33	L04	KY800510		
**UR00280 (SZU)**	Austria, Salzburg	*Lecidea* sp. UR00280	LP_Lcd34	L04	MK620250		
UCR_43266UCR1	USA, California	*L. laboriosa* Müll. Arg.	LP_Lcd35	L04	EU259902	MK684890	
**AAS_Convey00507B**	Antarctica, Ellsworth Land	*L. silacea* Ach.	LP_Lcd36	L05	MK620077		
T42156 (SZU)	Austria, Tirol	*L. silacea* Ach.	LP_Lcd37	L05	GU074424	GU074496	
**UR00271 (SZU)**	Austria, Salzburg	*L. silacea* Ach.	LP_Lcd38	L05	MK620249		
**T48781(SZU)**	Antarctica, S. Victoria Land	*Lecidea* sp. T48781	LP_Lcd39	L05	MK620097		
UCR_646Kerry	USA, California	*L. fuscoatrina* Hertel & Leuckert	LP_Lcd41	L06	EU259900		MK684919
LD L03170	Sweden	*L. plana* (J. Lahm) Nyl.	LP_Lcd42	L07	EU259903	GU074497	MK684895
LD L03316	Sweden	*L. plana* (J. Lahm) Nyl.	LP_Lcd43	L07	EU259904	GU074498	MK684896
**MAF_LI_1c (SZU)**	Antarctica, Livingston Island	*L. lithophila* (Ach.) Ach.	LP_Lcd44	L08	MK620090		
**UR00246 (SZU)**	Austria, Oberösterreich	*L. lithophila* (Ach.) Ach.	LP_Lcd45	L08	MK620054	MK684743	MK685037
Leavitt 17.621 (BRY-C)	USA, Nevada	*Lecidea* 'sp. Sl17621'	LP_Lcd46	L09	MK990108		
T42730 (SZU)	Austria, Osttirol	*L. obluridata* Nyl.	LP_Lcd47	L09	GU074423		
	Norway	*L. leucothallina* Arnold	LP_Lcd48	L09	MG973072		
	China	*L. fuscoatra* (L.) Ach. var. *grisella* (Flörke) Nyl.	LP_Lcd49	L10	KR061350		
	Pakistan	*L. aptrootii* M. Khan, A.N. Khalid, H.T. Lumbsch	LP_Lcd49	L10	MH618901		
		L. uniformis McCune	LP_Lcd50	L10	KY800507		
T40136 (SZU)	Austria	*L. fuscoatra* (L.) Ach.	LP_Lcd51	L10	EU263922	GU074490	MK684909
**AAS_FFE00012**	Antarctica, Ellsworth Land	*L. cancriformis* (C.W. Dodge & G.E. Baker)	LP_Lcd58	L11	MK620078	MK684570	MK684892
**LE_A151501**	Antarctica, MacRobertson Land	*L. cancriformis* (C.W. Dodge & G.E. Baker)	LP_Lcd58	L11	MK620087	MK684576	MK684904
**LE_A151902**	Antarctica, MacRobertson Land	*L. cancriformis* (C.W. Dodge & G.E. Baker)	LP_Lcd58	L11	MK620088	MK684577	MK684905
LE_A057501	Antarctica, Princ. Elisabeth Land	*L. cancriformis* (C.W. Dodge & G.E. Baker)	LP_Lcd58	L11	GU074440	GU074479	
T42988 (SZU)	Antarctica, Darwin Area	*L. cancriformis* (C.W. Dodge & G.E. Baker)	LP_Lcd58	L11	GU074435	GU074481	MK684912
T35604 (SZU)	Antarctica, Darwin Area	*L. cancriformis* (C.W. Dodge & G.E. Baker)	LP_Lcd58	L11	EU257671	GU074480	

**Voucher_id**	**Country**	**Mycobiont species**	**OTU_id**	**Cluster**	**ITS**	**mtSSU**	**RBP1**
**T43005 (SZU)**	Antarctica, S. Victoria Land	*L. cancriformis* (C.W. Dodge & G.E. Baker)	LP_Lcd58	L11	MK620096	MK684578	MK684913
T43020 (SZU)	Antarctica, S. Victoria Land	*L. cancriformis* (C.W. Dodge & G.E. Baker)	LP_Lcd58	L11	GU074431	GU074484	MK684914
T43028 (SZU)	Antarctica, S. Victoria Land	*L. cancriformis* (C.W. Dodge & G.E. Baker)	LP_Lcd58	L11	GU074430		
**LE_A040202**	Antarctica, Princ. Elisabeth Land	*L. cancriformis* (C.W. Dodge & G.E. Baker)	LP_Lcd58	L11	MK620080		
**UR00372 (SZU)**	Austria, Salzburg	*L. lapicida* (Ach.) Ach.	LP_Lcd67	L14	MK620256		
**MAF_LI_2f (SZU)**	Antarctica, Livingston Island	*L. lapicida* (Ach.) Ach.	LP_Lcd68	L14	MK620093		
**UR00379 (SZU)**	Austria, Salzburg	*L. lapicida* (Ach.) Ach.	LP_Lcd68	L14	MK620257		
**UR00423 (SZU)**	Austria, Salzburg	*L. lapicida* (Ach.) Ach.	LP_Lcd69	L14	MK620261		
**MAF_LI_3b (SZU)**	Antarctica, Livingston Island	*L. lapicida* (Ach.) Ach.	LP_Lcd71	L14	MK620094		
**T48875 (SZU)**	Antarctica, S. Victoria Land	*L. lapicida* (Ach.) Ach.	LP_Lcd71	L14	MK620098		
**US12426a(SZU)**	Norway, Svalbard	*L. lapicida* (Ach.) Ach.	LP_Lcd72	L14	MK620269		
**US12398(SZU)**	Norway, Svalbard	*L. lapicida* (Ach.) Ach.	LP_Lcd73	L14	MK620264		
H42375 (GZU)	Austria	*L. lapicida* (Ach.) Ach. var. *pantherina* Ach.	LP_Lcd74	L14	AF332118		
T42143	Austria, Tirol	*L. lapicida* (Ach.) Ach. var. *pantherina* Ach.	LP_Lcd74	L14		GU074494	
**US12409(SZU)**	Norway, Svalbard	*L. lapicida* (Ach.) Ach.	LP_Lcd75	L14	MK620266		
**UR00418 (SZU)**	Austria, Salzburg	*L. lapicida* (Ach.) Ach. var. *lapicida* Ach.	LP_Lcd77	L14	MK620259		
T42745 (SZU)	Austria, Osttirol	*L. lapicida* (Ach.) Ach. var. *lapicida* Ach.	LP_Lcd77	L14	GU074425	GU074493	MK684910
**UR00355 (SZU)**	Austria, Salzburg	*L. lapicida* (Ach.) Ach. var. *lapicida* Ach.	LP_Lcd77	L14	MK620255		
**UR00289 (SZU)**	Austria, Salzburg	*L. medusula* (C.W. Dodge) Hertel	LP_Lcd78	L15	MK620251		
**US12432a(SZU)**	Norway, Svalbard	*L. medusula* (C.W. Dodge) Hertel	LP_Lcd78	L15	MK620270		
	USA, Alaska	*L. protobacina* Nyl.	LP_Lcd79	L16	KY800506		
**T48883b (SZU)**	Antarctica, S. Victoria Land	*Lecidea* sp. T48883b	LP_Lcd80	L17	MK620099		
T43426	Norway, Svalbard	*L. auriculata* Th.Fr.	LP_Lcd81a	L18	GU074428	GU074499	MK684916
LD_L05184	Sweden	*L. auriculata* Th.Fr.	LP_Lcd82	L18	GU074427	GU074500	
**US12411(SZU)**	Norway, Svalbard	*L. auriculata* Th.Fr.	LP_Lcd83	L18	MK620267		
H49249 (GZU)	Austria, Steiermark	*L. tesselata* Flörke	LP_Lcd85	L18	EU263926	GU074491	MK684894
	USA, North Carolina	*Porpidia albocaerulescens* (Wulfen) Hertel & Knoph	LP_PorP01	P01	KJ653476	KJ653472	DQ986828
	USA, West Virginia	*P. degelii* (H. Magnusson) Lendemer	LP_Porp02	P01	KJ653480	KJ653474	
	Faroe Islands	*P. rugosa* (Taylor) Coppins & Fryday	LP_Porp03	P01	KJ162320		
	UK, Wales	*P. hydrophila* (Fr.) Hertel & A.J.Schwab	LP_Porp04	P02	KJ162319		
	Norway	*P. flavicunda* (Ach.) Gowan	LP_Porp05	P03	KJ162332		
	Norway, Swalbard	*P. flavicunda* (Ach.) Gowan	LP_Porp05	P03	KP314488		
**US12413(SZU)**	Norway, Swalbard	*P. flavicunda* (Ach.) Gowan	LP_Porp05	P03	MK620268		
**US12432c(SZU)**	Norway, Swalbard	*P. flavicunda* (Ach.) Gowan	LP_Porp05	P03	MK620271		
	Norway	*P. melinodes* (Körb.) Gowan & Ahti	LP_Porp06	P03	KJ162326		
	China	*P. speirea* (Ach.) Kremp	LP_Porp07	P03	KY509522		
	China	*P. speirea* (Ach.) Kremp	LP_Porp07	P03	KY509523		
	Ireland	*P. tuberculosa* (Sm.) Hertel & Knoph	LP_Porp08	P03	KJ162322		
**MAF_LI_1a (SZU)**	Antarctica, Livingston Island	inc. sed. *Porpidia* sp. 1	LP_Porp10	P05	MK620089		
**UR00296 (SZU)**	Austria, Salzburg	inc. sed. *Porpidia* sp. 1	LP_Porp10	P05	MK620253		
**UR00428 (SZU)**	Austria, Salzburg	inc. sed. *Porpidia* sp. 1	LP_Porp10	P05	MK620262		
	Austria, Tirol	*P. contraponenda* (Arnold) Knoph & Hertel	LP_Porp13	P05	KJ162298		

Voucher_id	Country	Mycobiont species	OTU_id	Cluster	ITS	mtSSU	RBP1
	Ireland	*P. cineroatra* (Ach.) Hertel & Knoph	LP_Porp14	P05	KJ162306	KF683104	
	Turkey	*P. musiva* (Körb.) Hertel & Knoph	LP_Porp15	P05	HQ605939		
	UK, Wales	*P. irrigua* A. Orange	LP_Porp16	P05	KJ162302		
**UR00248 (SZU)**	Austria, Oberösterreich	*Porpidia* sp. UR00248	LP_Porp17	P05	MK620248		
	UK, Wales	*P. striata* Fryday	LP_Porp18	P06	KJ162316		
	Faroe Islands	*P. islandica* Fryday	LP_Porp19	P07	KJ162313		
**UR00411(SZU)**	Austria, Salzburg	*P. macrocarpa* (DC.) Hertel & A.J.Schwab	LP_Porp20	P08	MK620258	MK684730	MK685056
**MAF_LI_4b (SZU)**	Antarctica, Livingston Island	*P. macrocarpa* (DC.) Hertel & A.J.Schwab	LP_Porp21	P08	MK620095		
T39740 (SZU)	Austria	*P. macrocarpa* (DC.) Hertel & A.J.Schwab	LP_Porp22	P08	EU263923	GU074511	
	UK, Wales	*P. macrocarpa* (DC.) Hertel & A.J.Schwab	LP_Porp23	P08	KJ162271		
**UR00292 (SZU)**	Austria, Salzburg	*P. macrocarpa* (DC.) Hertel & A.J.Schwab	LP_Porp26	P08	MK620252		
	Norway	*P. flavocruenta* Fryday & Buschbom	LP_Porp27	P08	KJ162274		
**MAF_LI_2c (SZU)**	Antarctica, Livingston Island	*P.* aff. *macrocarpa* (DC.) Hertel & A.J.Schwab	LP_Porp28	P08	MK620092		
**UR00330 (SZU)**	Austria, Salzburg	*P.* aff. *macrocarpa* (DC.) Hertel & A.J.Schwab	LP_Porp28	P08	MK620254		
	Turkey, Trabazon	*P. crustulata* (Ach.) Hertel & Knoph	LP_Porp29	P08	HQ605941		
	China, Guancen Mountain	*P. crustulata* (Ach.) Hertel & Knoph	LP_Porp30	P08	KF303294		
	USA, Washington	*Cyclohymenia epilithica* McCune & M.J.Curtis	Cyc01	C01	KY800503		
	USA, Oregon	*Cyclohymenia epilithica* McCune & M.J.Curtis	Cyc01	C01	KY800505		
T39660 (SZU)	Austria, Salzburg	*Farnoldia jurana* (Schaer.) Hertel ssp. *jurana*	F01		EU263920	GU074511	MK684889
T35647 (SZU)	Antarctica, Darwin Area	*Carbonea vorticosa* (Flörke) Hertel			JN873866		
T43429 (SZU)	Norway, Svalbard	*C. vorticosa* (Flörke) Hertel			JN873870		
T44628 (SZU)	Antarctica, Darwin Area	*Lecanora fuscobrunnea* C.W. Dodge & G.E. Baker			JN873875		
UCR_47815UCR1	USA, California	*L. polytropa* (Ehrh. ex Hoffm.) Rabenh.			JN873881		
T33597 (SZU)	Antarctica, S. Victoria Land	*Rhizoplaca macleanii* (Dodge) Castello			JN873905		
T38107 (SZU)	Switzerland, Wallis	*R. melanophthalma* (DC.) Leuckert & Poelt			JN873910		

S1-2d Cultured *Trebouxia* specimen provided by Sieglinde Ott, Heinrich Heine Universität, Düsseldorf, Germany

Culture	Country	Mycobiont species	Cultured photobiont			Accession number		
			Species	Clade	OTU_id	ITS	psbJ-L	COX2
1_SO	Antarctica, Southern Maritime Antarctica	*Umbilicaria antarctica* (Frey & I.M.Lamb) Llano	*Trebouxia* sp.	S	Tr_S02	MH813466	MH818849	MH818853
2_SO	Antarctica, Northern Victoria Land	*Pleopsidium chlorophanum* (Wahlenb.) Zopf	*Trebouxia* sp.	S	Tr_S02	MH813467	MH818850	MH818854
3_SO	Antarctica, Northern Victoria Land	*Buellia frigida* Darb	*Trebouxia* sp.	A	Tr_A02	MH813468	MH818851	MH818855
4_SO	Sweden	*Fulgensia bracteata* (Hoffm.) Räsenen	*Trebouxia* sp.	A	Tr_A12	MH813469	MH818852	MH818856

References

Fryday, A.M. & Hertel, H. (2014) A contribution to the family Lecideaceae s. lat. (Lecanoromycetidae inc. sed., lichenized Ascomycota) in the southern subpolar region; including eight new species and some revised generic circumscriptions. *The Lichenologist* **46**, 389-412.

Hafellner, J. & Türk, R. (2016) *The lichenized fungi of Austria – a new checklist of the taxa so far recorded, with data to distribution and substrate ecology*. Stapfia

Leavitt, S.D., Kraichak, E., Nelsen, M.P., Altermann, S., Divakar, P.K., Alors, D., Esslinger, T.L., Crespo, A. & Lumbsch, T. (2015) Fungal specificity and selectivity for algae play a major role in determining lichen partnerships across diverse ecogeographic regions in the lichen-forming family Parmeliaceae (Ascomycota). *Molecular Ecology* **24**, 3779-3797.

McCune, B., Curtis, M.J. & Di Meglio, J. (2017) New taxa and a case of ephemeral spore production in Lecideaceae from western North America. *Bryologist* **120**, 115-124.

Moya, P., Skaloud, P., Chiva, S., Garcia-Breijo, F.J., Reig-Arminana, J., Vancurova, L. & Barreno, E. (2015) Molecular phylogeny and ultrastructure of the lichen microalga *Asterochloris mediterranea* sp nov from Mediterranean and Canary Islands ecosystems. *International Journal of Systematic and Evolutionary Microbiology* **65**, 1838-1854.

Orange, A. (2014) *Porpidia irrigua*, a new species related to *P. contraponenda*. *Lichenologist* **46**, 269-284.

Ruprecht, U., Lumbsch, H.T., Brunauer, G., Green, T.G.A. & Turk, R. (2010) Diversity of *Lecidea* (Lecideaceae, Ascomycota) species revealed by molecular data and morphological characters. *Antarctic Science* **22**, 727-741.

Ruprecht U, Lumbsch HT, Brunauer G, Green TGA and Türk R (2012*b*) Insights into the diversity of *Lecanoraceae* (*Lecanorales*, Ascomycota) in continental Antarctica (Ross Sea region). *Nova Hedwigia* **94**, 287–306.

Ruprecht, U., Søchting, U. & Türk, R. (2016) *Porpidia navarina*, a new endemic species from Isla Navarino (southern Tierra del Fuego, Chile). *Herzogia* **29**, 596-609.

Skaloud, P., Steinova, J., Ridka, T., Vancurova, L. & Peksa, O. (2015) Assembling the Challenging Puzzle of Algal Biodiversity: Species Delimitation within the Genus Asterochloris (Trebouxiophyceae, Chlorophyta). *Journal of Phycology* **51**, 507-527.

Vancurova, L., Peksa, O., Nemcova, Y. & Skaloud, P. (2015) *Vulcanochloris* (Trebouxiales, Trebouxiophyceae), a new genus of lichen photobiont from La Palma, Canary Islands, Spain. *Phytotaxa* **219**, 118-132.

Wirth, V., Hauck, M. & Schultz, M. (2013) *Die Flechten Deutschlands*. Ulmer, Stuttgart (Hohenheim).

Zhao, X., Zhang, L.L., Zhao, Z.T., Wang, W.C., Leavitt, S.D. & Lumbsch, H.T. (2015) A Molecular Phylogeny of the Lichen Genus *Lecidella* Focusing on Species from Mainland China. *Plos One* **10**.

S1-3 Markers and primers including the respective melting temperature (Temp.) used in this study.

Marker	Primer Name	Organism	Primer Sequence 5'-> 3'	Temp. C°	Reference
ITS	18S-ITS uni-for	*Trebouxia*	gtg aac ctg cgg aag gat cat t	60.3	(Ruprecht *et al.* 2012)
	ITS1	Mycobiont general	tcc gta ggt gaa cct gcg g	61.0	(White *et al.* 1990)
	ITS1aT	*Trebouxia*	atc tat cgt gxm mac acc g	54.5	(Ruprecht *et al.* 2014)
	ITS1F	Mycobiont general	ctt ggt cat tta gag gaa gta a	54.7	(Gardes & Bruns 1993)
	ITS1L	Mycobiont general	gta ggt gaa cct gcg gaa	56.0	this study
	ITS1-sense-A	*Asterochloris*	tcc aca ccg agm aca ac	54.0	(Ruprecht *et al.* 2014)
	ITS1T	*Trebouxia*	gga agg atc att gaa tct atc gt	57.1	(Kroken & Taylor 2000)
	ITS2-antisense-A	*Asterochloris*	aag gtt tcc ctg ctt gac a	54.5	(Ruprecht *et al.* 2014)
	ITS4	Mycobiont general	tcc tcc gct tat tga tat gc	55.3	(White *et al.* 1990)
	ITS4bT_mod	*Trebouxia*	cca aaa sgc gtc ctg ca	55.2	(Ruprecht *et al.* 2014)
	ITS4L	Mycobiont general	cga tcc gag gtc aac ca	55.2	this study
	ITS4T	*Trebouxia*	gtt cgc tcg ccg cta cta	58.2	(Kroken & Taylor 2000)
	LR1850	*Chlorella*	cct cac ggt act tgt tc	50.4	(Bhattacharya *et al.* 1996)
	nr-SSU-1780-5'	*Asterochloris*	tgc gga agg atc att gat tc	55.3	(Piercey-Normore & Depriest 2001)
	NS7m	*Chlorella*	ggc aat aac agg tct gt	52.8	(Bhattacharya *et al.* 1996)
RPB1	fRPB1-C rev	*Lecidea, Porpidia, Poeltidea*	ccx gcd atx tcr ttr tcc atr ta	56.8	(Matheny *et al.* 2002)
	gRPB1-A for		gak tgt cck ggw cat ttt gg	57.3	(Matheny *et al.* 2002)
	RPB1_for_Lec		cca gtc tty cay gtt ggt at	55.3	this study
mtSSU	CU6	*Lecidea, Porpidia, Poeltidea*	tgt ggc acg tct ata gcc ca	59.4	(https://nature.berkeley.edu/brunslab/tour/primers.html)
	mrSSU1		agc agt gag gaa tat tgg tc	55.3	(Zoller *et al.* 1999)
	mtSSU for2		aat xgc ctr rcg gck gaa c	55.3	(Ruprecht *et al.* 2010)
	mtSSU rev2		aaa ata aag taa ggg ytt rcg tta att	55.9	(Ruprecht *et al.* 2010)
COX2	COXIIf2	*Trebouxia*	tta acg cct aac gag gga ac	57.3	(Lindgren *et al.* 2014)
	COXIIr		atacgaaatcccgttcctga	55.3	(Lindgren *et al.* 2014)
	COX sense		aacgcctaacgagggaact	56.7	this study
psbJ-L	psbF	*Trebouxia*	gtw gtw cca gta ttr gac at	52.2	(Werth & Sork 2010)
	psbR		aac cra atc cax aya aac aa	50.1	(Werth & Sork 2010)
	psbL-sense		tta att ttc gtt tta gct gtt c	50.9	(Ruprecht *et al.* 2014)
	psbJ-antisense		ttc cta aat ttt ttc gtt tca ata	50.8	(Ruprecht *et al.* 2014)

References

Bhattacharya, D., Friedl, T., Damberger, S. (1996) Nuclear-encoded rDNA group I introns: Origin and phylogenetic relationships of insertion site lineages in the green algae. *Molecular Biology and Evololution* **13**, 978-989.

Gardes, M., Bruns, T. D. (1993) ITS Primers with Enhanced Specificity for Basidiomycetes - Application to the Identification of Mycorrhizae and Rusts. *Mol Ecol* **2**, 113-118.

Kroken, S., Taylor, J. W. (2000) Phylogenetic species, reproductive mode, and specificity of the green alga *Trebouxia* forming lichens with the fungal genus *Letharia*. *Bryologist* **103**, 645-660.

Lindgren, H., Velmala, S., Hognabba, F., Goward, T., Holien, H., Myllys, L. (2014) High fungal selectivity for algal symbionts in the genus Bryoria. *Lichenologist* **46**, 681-695.

Matheny, P. B., Liu, Y. J. J., Ammirati, J. F., Hall, B. D. (2002) Using RPB1 sequences to improve phylogenetic inference among mushrooms (Inocybe, Agaricales). *American Journal of Botany* **89**, 688-698.

Piercey-Normore, M. D., Depriest, P. T. (2001) Algal switching among lichen symbioses. *American Journal of Botany* **88**, 1490-1498.

Ruprecht, U., Lumbsch, H. T., Brunauer, G., Green, T. G. A., Türk, R. (2010) Diversity of *Lecidea* (Lecideaceae, Ascomycota) species revealed by molecular data and morphological characters. *Antarcic Science* **22**, 727-741.

Ruprecht, U., Brunauer, G., Printzen, C. (2012) Genetic diversity of photobionts in Antarctic lecideoid lichens from an ecological viewpoint. *Lichenologist* **44**, 661-678.

Ruprecht, U., Brunauer, G., Türk, R. (2014) High photobiont diversity in the common European soil crust lichen *Psora decipiens*. *Biodiversity and conservation* **23**, 1771-1785.

Werth, S., Cornejo, C., Scheidegger, C. (2010) A species-specific real-time PCR assay for identification of three lichen-forming fungi, Lobaria pulmonaria, Lobaria immixta and Lobaria macaronesica. *Molecular ecology resources* **10**, 401-403.

White, T. J., Bruns, T. D., Lee, S. B., Taylor, J. W. (1990) Amplification and direct sequencing of fungal ribosomal genes for phylogenies. *Academic Press*, *NY.*

Zoller, S., Scheidegger, C., Sperisen, C. (1999) PCR primers for the amplification of mitochondrial small subunit ribosomal DNA of lichen-forming ascomycetes. *Lichenologist* **31**, 511-516.

S1-4 Evolutionary models of the Maximum Likelihood analyses.

Phylogeny	Mycobiont	Photobiont
Lecidea/Porpidia/Poeltidea - ITS	TIM2+F+I+G4	
Lecidea/Porpidia/Poeltidea - ITS/mtSSU/*RPB1*	TIM2e+I+G4	
Lecidella - ITS	TNe+I+G4	
Asterochloris - ITS		TIM2e+I+G4
Trebouxia - ITS		SYM+I+G4
Trebouxia - ITS/psbJ-L/COX2		TPM3u+F+G4

References

Kalyaanamoorthy, S., Minh, B.Q., Wong, T.K.F., von Haeseler, A. & Jermiin, L.S. (2017) ModelFinder: fast model selection for accurate phylogenetic estimates. *Nature Methods* **14**, 587-+.

S1-5 Sequence similarity (%) of OTUs with more than 3 accessions per OTU (ITS)

OTU ≥ 3 accessions	sub - OTU	sequence similarity %
LP_Lcd01 (7)		99.0
LP_Lcd05 (3)		99.4
LP_Lcd09 total (7)		97.3
	LP_09a (5)	97.9
	LP_09b (2)	99.8
LP_Lcd10 (6)		99.4
LP_Lcd18 (6)		98.6
LP_Lcd25 (3)		97.8
LP_Lcd27 (3)		99.2
LP_Lcd54 (3)		99.1
LP_Lcd57 (3)		99.2
LP_Lcd58 (7)		98.6
LP_Lcd66 (8)		98.7
LP_Lcd70 (7)		98.8
LP_Lcd77 (3)		98.9
LP_Lcd78 (4)		99.0
LP_Lcd81 total (7)		94.8
	LP_Lcd81a (1)	-
	LP_Lcd81b (6)	98.8
LP_Lcd84 (4)		98.8
LP_Porp05 (4)		99.6
LP_Porp10 (4)		98.8
LP_Porp20 (4)		98.5
LL_N01 (3)		99.4
Tr_A02 (6)		99.4
Tr_A04 total (8)		96.7
	Tr_A04a (5)	97.7
	Tr_A04b (3)	98.8
	Tr_I01i (6)	97.5
	Tr_I01j (7)	98.6
Tr_I17 (4)		99.1
Tr_S02 total (17)		94.8
	Tr_S02 (12)	98.0
	Tr_S02b (3)	98.4
Tr_S07 (5)		99.1
Tr_S20 (3)		98.8

References

Leavitt, S.D., Kraichak, E., Nelsen, M.P., Altermann, S., Divakar, P.K., Alors, D., Esslinger, T.L., Crespo, A. & Lumbsch, T. (2015) Fungal specificity and selectivity for algae play a major role in determining lichen partnerships across diverse ecogeographic regions in the lichen-forming family Parmeliaceae (Ascomycota). *Molecular Ecology* **24**, 3779-3797.

Puillandre, N., Lambert, A., Brouillet, S. & Achaz, G. (2012) ABGD, Automatic Barcode Gap Discovery for primary species delimitation. *Molecular Ecology* **21**, 1864-1877.
