# Supplementary material for: High levels of endemism and local differentiation in the fungal and algal symbionts of saxicolous lecideoid lichens along a latitudinal gradient in southern South America
Source: Lichenologist (Lond). 2020 Jul 29;52(4):287–303. doi: 10.1017/S0024282920000225 (PMC7396322; doi:10.1017/S0024282920000225)
Supplement: Supplementary file 1 [file S0024282920000225sup001.zip › S0024282920000225sup001/S2_150520.pdf]

Supplementary Material S2

Table of Contents

S2-1a…………………… 1
S2-1b…………………… 4
S2-2…………………….. 5
S2-3…………………….. 6

S2-1a Voucher ID (bold italic, sSA specimen; bold, new sequences) and accession numbers including published species names (*Lecidea, Porpidia*, *Poeltidea*) assigned to the respective OTUs, biogeographic distribution (NH, Northern Hemisphere; C, cosmopolitan; sSA, southern South America; Ant, Antarctica), species/groups and clusters as shown in Figure S1-1b.

OTU	Voucher-id, published species names	Acc. Num.	Distribution	Species - Groups	Cluster
LP_Lcd01	**AAS_Convey00458**	MK620076			
LP_Lcd01	**AAS_Smith12030**	MK620079			
LP_Lcd01	LE_A060124, *L. atrobrunnea*	GU074457	C		
LP_Lcd01	***UR00200***	KX120206			
LP_Lcd01	***UR00235***	MK620240			
LP_Lcd01	*L. syncarpa*	MK990107			
LP_Lcd02	*L. syncarpa*	KF570277	NH		
LP_Lcd03	UCR174803, *L. atrobrunnea* subsp. *saxosa*	GU074456	NH	*L. atrobrunnea* - clade	
LP_Lcd04	Lund_LG770, *L. atrobrunnea*	EU259897	NH		
LP_Lcd05	**LE_A060137**	MK620083			
LP_Lcd05	***UR00001***	MK620100	sSA / Ant		
LP_Lcd05	***UR00212***	KX120207			
LP_Lcd06	**US12406**	MK620265	NH		
LP_Lcd07	*L. atrobrunnea*	MK990105	NH		
LP_Lcd08	T34435, *L. atrobrunnea* subsp. *stictica*	EU259899	NH		
LP_Lcd09a	***UR00196***	KX120208			L01
LP_Lcd09a	***UR00202***	MK620211			
LP_Lcd09a	***UR00216***	MK620224	sSA		
LP_Lcd09a	***UR00217***	MK620225		*Lecidea* sp. 1	
LP_Lcd09a	***UR00222***	MK620230			
LP_Lcd09b	***UR00233***	MK620238			
LP_Lcd09b	***UR00236***	MK620241	sSA		
LP_Lcd10	***UR00129***	KX120205			
LP_Lcd10	***UR00150***	MK620179	sSA		
LP_Lcd10	***UR00152***	MK620181			
LP_Lcd10	***UR00151***	MK620180		*L. promiscens*	
LP_Lcd11	***UR00081***	MK620137	sSA		
LP_Lcd12	***UR00149***	MK620178	sSA		
LP_Lcd13	**UR00471**	MK620263	NH		
LP_Lcd14	**MAF_LI_2a**	MK620091	Ant		
LP_Lcd15	**UR00422**	MK620260	NH	*L. swartzioidea*	
LP_Lcd16	T39641, *L. confluens*	EU263921	NH	*L. confluens*	
LP_Lcd17	*P. speirea*	HQ650631	NH	*L.* sp.	L02
LP_Lcd18	***UR00177***	KX120211			
LP_Lcd18	***UR00242***	MK620243			
LP_Lcd18	**LE_A111504**	MK620085	C	*Lecidea* sp. 2	L03
LP_Lcd18	***UR00158***	MK620187			
LP_Lcd18	***UR00194***	MK620207			
LP_Lcd18	T43427, *L.* sp. T43427	GU074426			
LP_Lcd19	*L. polypycnidophora 'B'*	MK990117	NH		
LP_Lcd20	*L.* '*andersonii* agg. sp 3'	MK990110	NH		
LP_Lcd21	LOSD #1165	EU263929	NH		
LP_Lcd21	*L. polypycnidophora* 'B'	MH231446			
LP_Lcd22	**LE_A0511701,** *L. polypycnidophora*	MK620081	Ant	*L. andersonii* - complex	
LP_Lcd23	*L.* '*andersonii* agg. sp 2'	MK990111	NH		
LP_Lcd24	*L.* '*andersonii* agg. sp 1'	MK990109	NH		
LP_Lcd25	LE_A0515601, *L. andersonii*	GU074445			
LP_Lcd25	T43425, *L. andersonii*	GU074442	C		
LP_Lcd25	*L. andersonii*	MK990112			
LP_Lcd26	**LE_A133003,** *L.* sp. UCR1	MK620086	Ant		
LP_Lcd27		MK990123			L04
LP_Lcd27	*UR00218*	MK620226	C		
LP_Lcd27	*UR00195*	KX120210			
LP_Lcd28		MK990126	NH		
LP_Lcd29		MK990121	C	*Lecidea* sp. 3	
LP_Lcd29	***UR00135***	KX120209			
LP_Lcd30		MK990118	NH		
LP_Lcd31		MK990115	NH		
LP_Lcd32		MK990116	NH		
LP_Lcd33	*L. violascens*	KY800510	NH	*L. violascens*	
LP_Lcd34	**UR00280**	MK620250	NH		
LP_Lcd35	UCR_43266UCR1, *L. laboriosa*	EU259902	NH	*L. laboriosa*	
LP_Lcd36	**AAS_Convey00507B**	MK620077	Ant		
LP_Lcd37	T42156	GU074424	NH	*L. silacea* - clade	
LP_Lcd38	**UR00271**	MK620249	NH		L05
LP_Lcd39	**T48781**	MK620097	Ant		
LP_Lcd40	***UR00130***	MK620164	sSA		
LP_Lcd41	UCR_646Kerry	EU259900	NH	*L. fuscoatrina*	L06
LP_Lcd42	LD L03170, *L. plana*	EU259903	NH	*L. plana*	L07
LP_Lcd43	LD L03316, *L. plana*	EU259904	NH		
LP_Lcd44	**MAF_LI_1c**	MK620090	Ant	*L. lithophila*	L08
LP_Lcd45	**UR00246**	MK620054	NH		
LP_Lcd46	*L.* 'sp. Sl17621'	MK990108	NH		
LP_Lcd47	T42730, *L. obluridata*	GU074423	NH	*L. obluridata*	L09
LP_Lcd48	*L. leucothallina*	MG973072	NH	*L. leucothallina*	

LP_Lcd49	*L. fuscoatra* var. *grisella*	KR061350	NH		
LP_Lcd49	*L. aptrootii*	MH618901			
LP_Lcd50	*L. uniformis*	KY800507	NH	*L. fuscoatra* - complex	L10
LP_Lcd51	T40136, *L. fuscoatra*	EU263922	NH		
LP_Lcd52	***UR00179***	MK620198	sSa		
LP_Lcd52	***UR00190***	MK620205			
LP_Lcd53	***UR00099***	MK620147	sSa		
LP_Lcd54	***UR00110***	MK620153			
LP_Lcd54	***UR00189***	MK620204	sSA		
LP_Lcd54	***UR00220***	MK620228			
LP_Lcd55	***UR00124***	MK620160	sSA		
LP_Lcd56	***UR00156***	MK620185	sSA		
LP_Lcd57	***UR00134***	MK620167			
LP_Lcd57	***UR00147***	MK620176	sSA		
LP_Lcd57	***UR00154***	MK620183		*L. cancriformis* - clade	L11
LP_Lcd58	**AAS_FFE00012**	MK620078			
LP_Lcd58	**LE_A151902**	MK620088			
LP_Lcd58	T35604, *L. cancriformis*	EU257671			
LP_Lcd58	T43020, *L. cancriformis*	GU074431	Ant		
LP_Lcd58	T43028, *L. cancriformis*	GU074430			
LP_Lcd58	**LE_A040202**	MK620080			
LP_Lcd58	T42988, *L. cancriformis*	GU074435			
LP_Lcd59	***UR00005***	MK659868	sSA		
LP_Lcd60	***UR00148***	MK620177	sSA		
LP_Lcd61	***UR00025***	MK620105	sSA		
LP_Lcd62	***UR00131***	MK620165	sSA		
LP_Lcd63	***UR00143***	MK620172	sSA	*Lecidea* sp. 4 - clade	L12
LP_Lcd63	***UR00144***	MK620173			
LP_Lcd64	***UR00122***	MK620159	sSA		
LP_Lcd65	***UR00096***	MK620145	sSA		L13
LP_Lcd66	***UR00073***	MK620131			
LP_Lcd66	***UR00114***	MK620155			
LP_Lcd66	***UR00186***	MK620202			
LP_Lcd66	***UR00008***	KX120202	sSA		
LP_Lcd66	***UR00009***	MK620103			
LP_Lcd66	***UR00094***	MK620143			
LP_Lcd66	***UR00105***	MK620150			
LP_Lcd66	***UR00091***	MK620142			
LP_Lcd67	**UR00372**	MK620256	sSA		
LP_Lcd68	**MAF_LI_2f**	MK620093	C		
LP_Lcd68	**UR00379**	MK620257			
LP_Lcd69	**UR00423**	MK620261	NH		
LP_Lcd70	***UR00002***	KX120200			
LP_Lcd70	***UR00055***	MK620118			
LP_Lcd70	***UR00059***	MK620121		*L. lapicida* - clade	L14
LP_Lcd70	***UR00017***	KX120201	sSA		
LP_Lcd70	***UR00077***	MK620135			
LP_Lcd70	***UR00084***	MK620139			
LP_Lcd70	***UR00228***	MK620233			
LP_Lcd71	**MAF_LI_3b**	MK620094	Ant		
LP_Lcd71	**T48875**	MK620098			
LP_Lcd72	**US12426a**	MK620269	NH		
LP_Lcd73	**US12398**	MK620264	NH		
LP_Lcd74	H42375, *L. lapicida* var. *pantherina*	AF332118	NH		
LP_Lcd75	**US12409**	MK620266	NH		
LP_Lcd76	***UR00232***	MK620237	sSA		
LP_Lcd77	**UR00418**	MK620259			
LP_Lcd77	T42745, *L. lapicida* var. *lapicida*	GU074425	NH		
LP_Lcd77	**UR00355**	MK620255			
LP_Lcd78	**UR00289**	MK620251			
LP_Lcd78	**US12432a**	MK620270	C	*L. medusula*	L15
LP_Lcd78	***UR00006***	KX120204			
LP_Lcd78	***UR00070***	MK620130			
LP_Lcd79	*L. protobacina*	KY800506	NH	*L. protobacina*	L16
LP_Lcd80	**T48883b**	MK620099	Ant		L17
LP_Lcd81a	T43426, *L. auricluata*	GU074428	NH		
LP_Lcd81b	***UR00010***	KX120212			
LP_Lcd81b	***UR00076***	MK620134			
LP_Lcd81b	***UR00062***	MK620124	sSA		
LP_Lcd81b	***UR00173***	MK620194		*L. auriculata* - clade	
LP_Lcd81b	***UR00175***	MK620196			
LP_Lcd81b	***UR00170***	MK620192			
LP_Lcd82	LD_L05184	GU074427	NH		L18
LP_Lcd83	**US12411**	MK620267	NH		
LP_Lcd84	***UR00141***	MK620170	sSA		
LP_Lcd85	***UR00138***	KX120213			
LP_Lcd85	***UR00139***	MK620169	C		
LP_Lcd85	H49249, *L. tesselata*	EU263926		*L. tesselata*	
LP_Lcd85	***UR00188***	MK620203			
LP_PorP01	*P. albocaerulescens*	KJ653476	NH	*P. albocaerulescens*	
LP_Porp02	*P. degelii*	KJ653480	NH	*P. degelii*	P01
LP_Porp03	*P. rugosa*	KJ162320	NH	*P. rugosa*	
LP_Porp04	*P. hydrophila*	KJ162319	NH	*P. hydrophila*	P02
LP_Porp05	*P. flavicunda*	KJ162332			
LP_Porp05		KP314488	NH	*P. flavicunda*	
LP_Porp05	**US12413**	MK620268			
LP_Porp05	**US12432c**	MK620271			P03
LP_Porp06	*P. melinodes*	KJ162326	NH	*P. melinodes*	
LP_Porp07	*P. speirea*	KY509522	NH	*P. speirea*	
LP_Porp07	*P. speirea*	KY509523			
LP_Porp08	*P. tuberculosa*	KJ162322	NH	*P. tuberculosa*	
Cyc01	*Cyclohymenia epilithica*	KY800503	NH	*C. epilithica*	C01
Cyc01		KY800505			
LP_Lcd86	***UR00075***	MK620133	sSA		
LP_Lcd86	***UR00098***	MK620146		*L. kalbii*	L19
LP_Lcd87	***UR00181***	MK620199	sSA		
LP_Porp09	***UR00013***	KX120214	sSA	*P. navarina*	P04
LP_Porp09	***UR00023***	KX120226			

LP_Porp10	**MAF_LI_1a**	MK620089	C	inc. sed. *Porpidia* sp. 1	P05
LP_Porp10	**UR00296**	MK620253			
LP_Porp10	**UR00428**	MK620262			
LP_Porp11	***UR00014***	KX120229	sSA		
LP_Porp12	***UR00012***	KX120227	sSA		
LP_Porp12	***UR00029***	KX120228			
LP_Porp13	*P. contraponenda*	KJ162298	NH	*P. contraponenda*	
LP_Porp14	*P. cineroatra*	KJ162306	NH	*P. cineroatra*	
LP_Porp15	*P. musiva*	HQ605939	NH	*P. musiva*	
LP_Porp16	*P. irrigua*	KJ162302	NH	*P. irrigua*	
LP_Porp17	**UR00248**	MK620248	NH		
LP_Porp18	*P. striata*	KJ162316	NH	*P. striata*	P06
LP_Porp19	*P. islandica*	KJ162313	NH	*P. islandica*	P07
LP_Porp20	***UR00022***	KX120232	C	*P. macrocarpa* - complex	P08
LP_Porp20	***UR00032***	KX120233			
LP_Porp20	***UR00027***	KX120234			
LP_Porp20	**UR00411**	MK620258			
LP_Porp21	**MAF_LI_4b**	MK620095	Ant		
LP_Porp22	T39740, *P. macrocarpa*	EU263923	NH		
LP_Porp23	*P. macrocarpa*	KJ162271	NH		
LP_Porp24	***UR00123***	KX120237	sSA		
LP_Porp25	***UR00193***	KX120236	sSA		
LP_Porp26	**UR00292**	MK620252	NH		
LP_Porp27	*P. flavocruenta*	KJ162274	NH		
LP_Porp28	**MAF_LI_2c**	MK620092	C		
LP_Porp28	**UR00330**	MK620254			
LP_Porp29	*P. crustulata*	HQ605941	NH	*P. crustulata*	
LP_Porp30	*P. crustulata*	KF303294	NH		
Poe01	***UR00026***	MK620106	sSA	*P. perusta* - clade	POE1
Poe02	***UR00037***	MK620111	sSA		POE2
Poe03	***UR00004***	MK620101	sSA	*Poeltidea* sp. 1	POE3

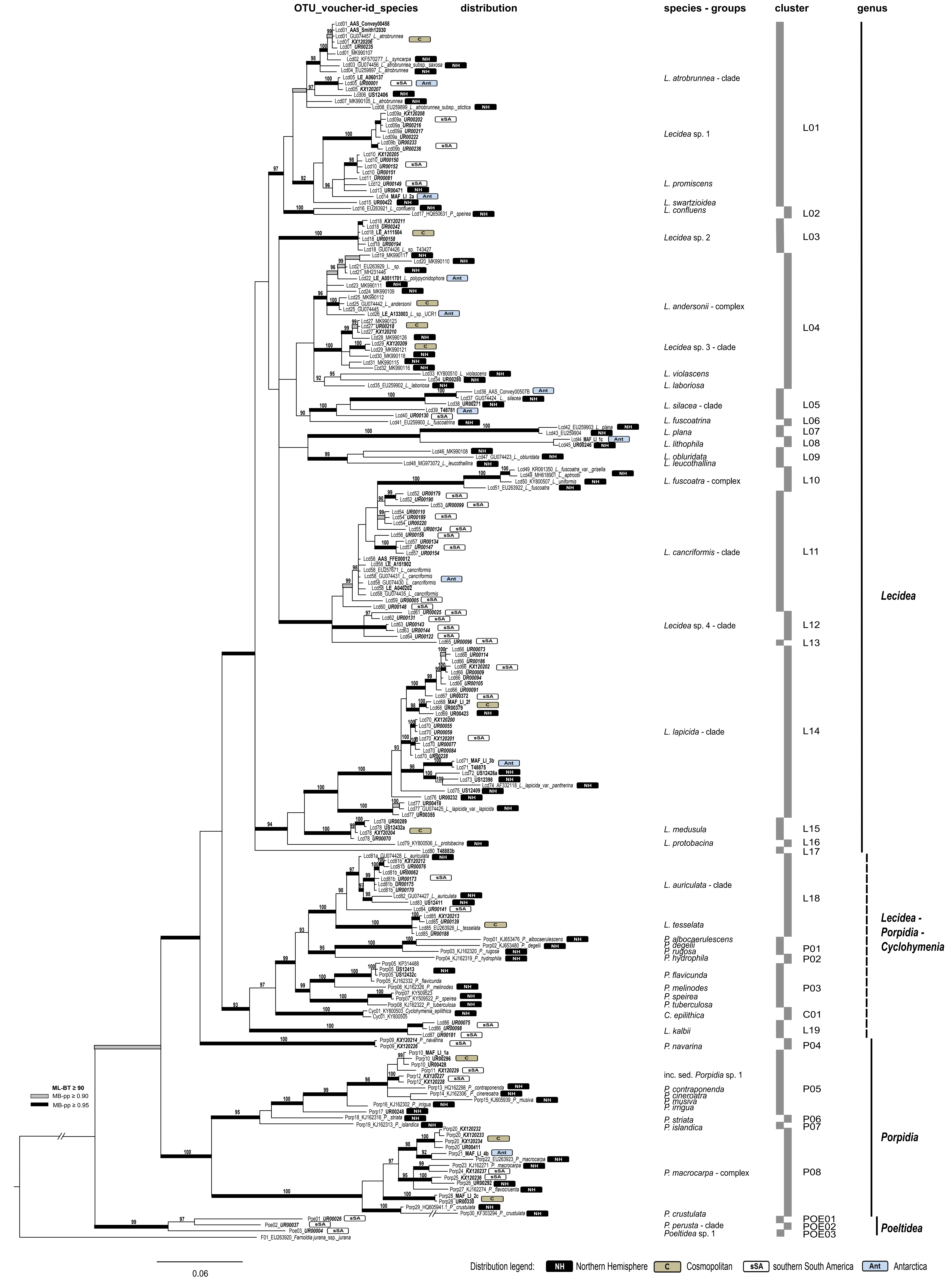

S2-1b: Phylogeny including all available relevant taxonomically identified sequences of the genera *Lecidea, Porpidia*, *Poeltidea* and *Cyclohymenia* using the marker ITS. OTU numbers predece voucher numbers and the published species names from Genbank, as well as the biogeographic distribution information for each OTU is included (NH, Northern Hemisphere; C, cosmopolitan; sSA, southern South America; Ant, Antarctica). The specimens from sSA are marked in bold italics and those from other parts of the world in bold. The vertical bars beside the phylogeny show the affiliation to clusters and genera. The bootstrap values with ≥ 95 support of ML analyses were directly mapped on the Bayesian tree with ≥ 0.90 (gray) and ≥ 0.95 (black) support posterior probability values (branches in bold).

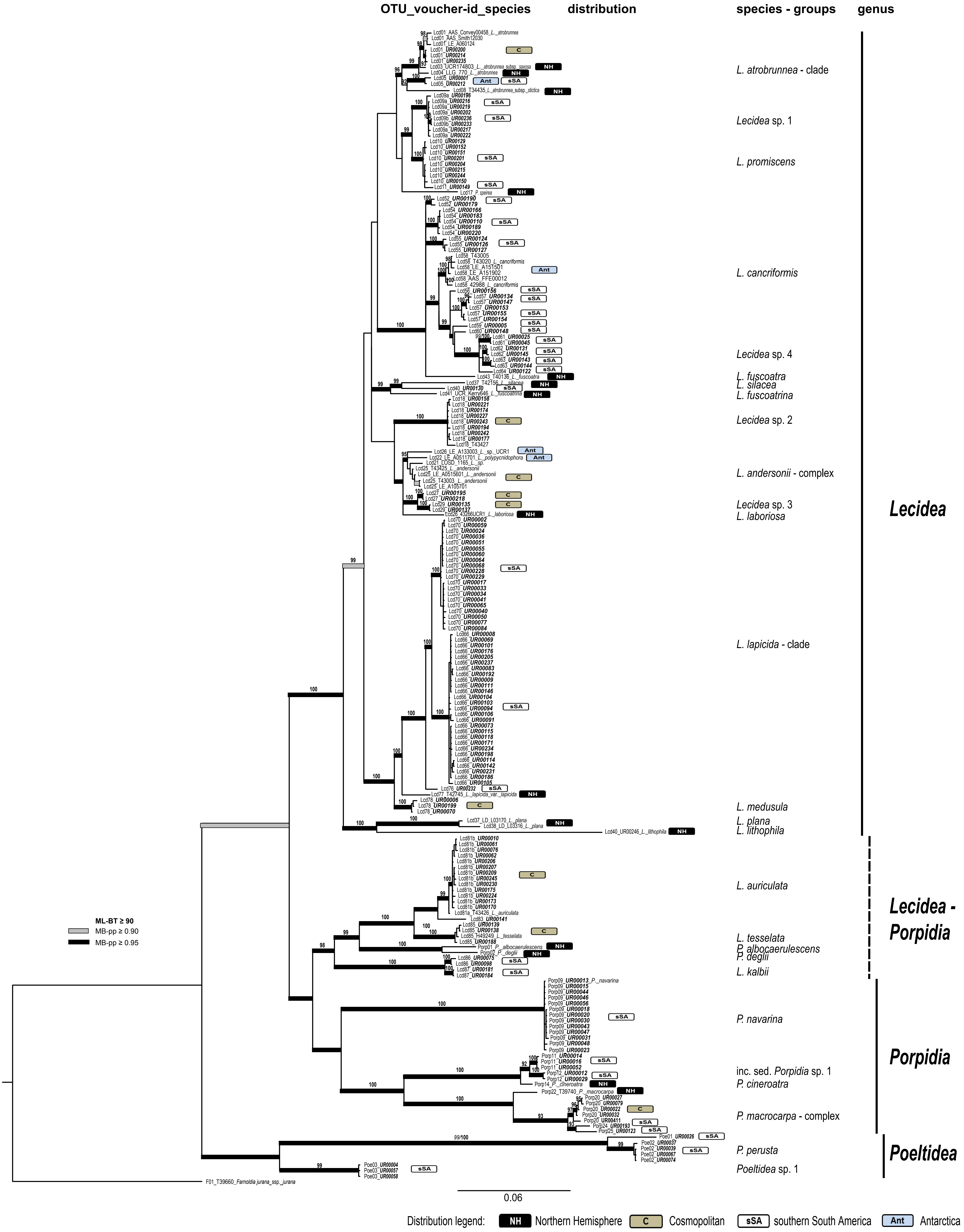

S2-2 Phylogeny of concatenated ITS, mtSSU and *RPB1* sequences including all investigated specimens from sSA, and additional accessions from Antarctica and Europe of the genera *Lecidea*, *Porpidia* and *Poeltidea*. OTU numbers precede voucher numbers and the published species names from Genbank as well as the biogeographic distribution information for each OTU is included (NH, Northern Hemisphere; C, cosmopolitan; sSA, southern South America; Ant, Antarctica). The specimen from sSA are marked in bold italics. The vertical bars beside the phylogeny show the affiliation to the genera. The bootstrap values with ≥ 95 support of ML analyses were directly mapped on the Bayesian tree with ≥ 0.90 (gray) and ≥ 0.95 (black) support posterior probability values (branches in bold).

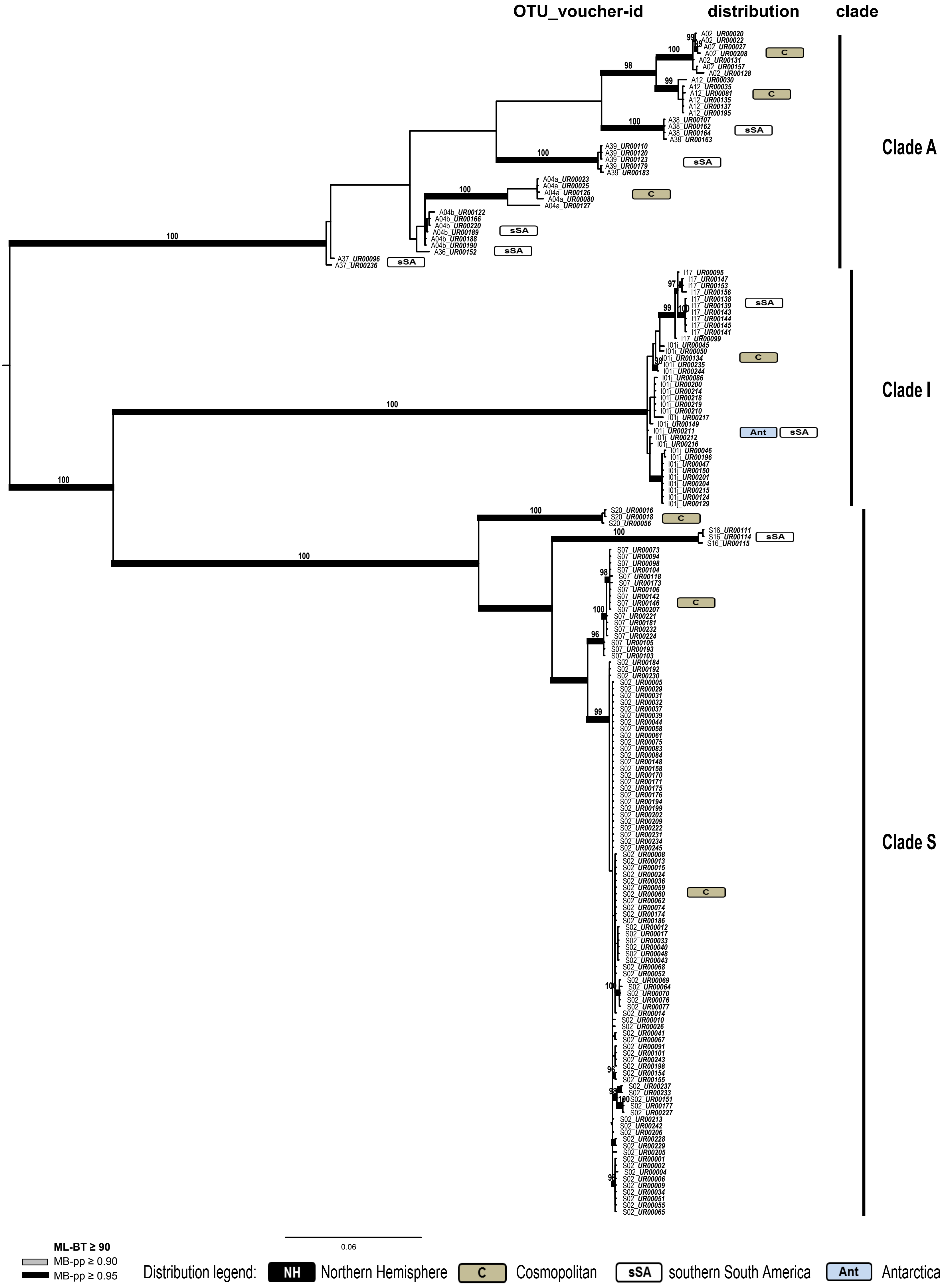

S2-3 Phylogeny of concatenated ITS, psbJ-L and COX2 sequences showing only the investigated specimens from sSA of the genus *Trebouxia*. OTU numbers precede voucher numbers and biogeographical distribution information for each OTU is included (NH, Northern Hemisphere; C, cosmopolitan; sSA, southern South America; Ant, Antarctica). The vertical bars beside the phylogeny show the affiliation to the clades and distribution. sSA accessions are highlighted in grey and those restricted to sSA on OTU-level, with a full-length bar. The bootstrap values with ≥ 95 support of ML analyses were directly mapped on the Bayesian tree with ≥ 0.90 (gray) and ≥ 0.95 (black)support posterior probability values (branches in bold).
